# Predicting Non-Alcoholic Steatohepatitis: A Lipidomics-Driven Machine Learning Approach

**DOI:** 10.3390/ijms25115965

**Published:** 2024-05-29

**Authors:** Thomai Mouskeftara, Georgios Kalopitas, Theodoros Liapikos, Konstantinos Arvanitakis, Georgios Germanidis, Helen Gika

**Affiliations:** 1 Laboratory of Forensic Medicine & Toxicology, School of Medicine, Faculty of Health Sciences, Aristotle University of Thessaloniki, 54124 Thessaloniki, Greece; mousthom@auth.gr; 2 Biomic AUTh, Center for Interdisciplinary Research and Innovation (CIRI-AUTH), Balkan Center B1.4, 10th km Thessaloniki-Thermi Rd., 57001 Thessaloniki, Greece; 3 Division of Gastroenterology and Hepatology, 1st Department of Internal Medicine, AHEPA University Hospital, School of Medicine, Faculty of Health Sciences, Aristotle University of Thessaloniki, 54636 Thessaloniki, Greece; gekalopi@auth.gr (G.K.); geogerm@auth.gr (G.G.); 4 Basic and Translational Research Unit, Special Unit for Biomedical Research and Education, School of Medicine, Faculty of Health Sciences, Aristotle University of Thessaloniki, 54636 Thessaloniki, Greece; 5 Laboratory of Hygiene, Social and Preventive Medicine and Medical Statistics, School of Medicine, Faculty of Health Sciences, Aristotle University of Thessaloniki, 54636 Thessaloniki, Greece; 6 Laboratory of Analytical Chemistry, Department of Chemistry, Aristotle University of Thessaloniki, 54124 Thessaloniki, Greece; theoliapikos@gmail.com; 7 First Department of Internal Medicine, AHEPA University Hospital, Aristotle University of Thessaloniki, 54636 Thessaloniki, Greece; arvanitak@auth.gr

**Keywords:** untargeted lipidomics, non-alcoholic fatty liver disease, plasma, machine learning

## Abstract

Nonalcoholic fatty liver disease (NAFLD), nowadays the most prevalent chronic liver disease in Western countries, is characterized by a variable phenotype ranging from steatosis to nonalcoholic steatohepatitis (NASH). Intracellular lipid accumulation is considered the hallmark of NAFLD and is associated with lipotoxicity and inflammation, as well as increased oxidative stress levels. In this study, a lipidomic approach was used to investigate the plasma lipidome of 12 NASH patients, 10 Nonalcoholic Fatty Liver (NAFL) patients, and 15 healthy controls, revealing significant alterations in lipid classes, such as glycerolipids and glycerophospholipids, as well as fatty acid compositions in the context of steatosis and steatohepatitis. A machine learning XGBoost algorithm identified a panel of 15 plasma biomarkers, including HOMA-IR, BMI, platelets count, LDL-c, ferritin, AST, FA 12:0, FA 18:3 ω3, FA 20:4 ω6/FA 20:5 ω3, CAR 4:0, LPC 20:4, LPC O-16:1, LPE 18:0, DG 18:1_18:2, and CE 20:4 for predicting steatohepatitis. This research offers insights into the connection between imbalanced lipid metabolism and the formation and progression of NAFL D, while also supporting previous research findings. Future studies on lipid metabolism could lead to new therapeutic approaches and enhanced risk assessment methods, as the shift from isolated steatosis to NASH is currently poorly understood.

## 1. Introduction

Nonalcoholic fatty liver disease (NAFLD) is nowadays a global public health problem [1], affecting more than 1 billion people worldwide [2,3]. The disease spectrum ranges from Nonalcoholic Fatty Liver (NAFL), which is characterized by simple steatosis with or without inflammation, to Non-Alcoholic Steatohepatitis (NASH), which is defined by the coexistence of steatosis, inflammation, and hepatocellular ballooning. Recently, due to the strong association of NAFLD/NASH with metabolic risk factors, such as metabolic syndrome (MetS), type 2 diabetes (T2DM), insulin resistance (IR) as well as obesity, metabolic dysfunction-associated steatotic liver disease (MASLD) has been proposed as the latest term to describe steatotic liver disease associated with MetS [4].

The molecular mechanisms in the pathogenesis of NAFL and its progression to NASH are poorly understood; however, the storage of lipid droplets inside hepatocytes as a result of intracellular lipid accumulation is considered the hallmark of NAFLD [5]. The accumulation derives from increased fatty acid absorption, increased de novo lipogenesis, and impaired fatty acid export and oxidation [6]. These cellular dysfunctions contribute to alterations in lipids’ homeostasis, leading to lipotoxicity [7,8]. A thorough investigation of the various lipid species along the different stages of NAFLD may provide important insights into the mechanisms underlying disease progression [9]. Up to today, liver biopsy remains the clinical gold standard for the definitive diagnosis. Nonetheless, this approach is notably invasive and expensive, posing potential risks of side effects and sampling errors. On the other hand, ultrasonography serves as a functional tool for diagnosing only fatty liver disease and not steatohepatitis, while the accuracy of this method is operator-dependent [10].

Extensive lipidomic studies have been conducted in both liver biopsies and blood plasma either on human subjects or mouse models to shed light on the biochemistry behind the progression of NAFLD but also towards the exploration of potential specific NASH biomarkers in blood [11]. A correlation between alterations in liver and blood lipidome during NAFLD progression is assumed since the liver is the primary organ of lipid metabolism and plasma lipids under fasting conditions primarily reflect the lipids excreted from this tissue [5]. Until now, plasma lipidomic analyses have revealed several lipid mediators, including fatty acids, sphingolipids, phospholipids, diacylglycerols, and triacylglycerols, as potential key contributors to the mechanism of disease progression toward NASH. Indeed, pro-inflammatory and pro-apoptotic factors are linked to increased concentrations of specific lipid species, such as saturated fatty acids and phospholipids, as well as to disruptions in ceramide-signaling or changes in cholesterol homeostasis [12]. However, it has not yet been established whether these modifications are reflected in the circulating lipids or whether NASH has a specific lipidomic profile [13].

For the non-invasive diagnosis of NASH, various diagnostic tests and numerous scores or indexes have been developed, incorporating clinical variables and/or plasma biomarkers to predict the presence of fibrosis [14], including the BARD score [15], the FIB-4 index [16], the fatty liver index (FLI) [17], the NAFLD fibrosis score (NFS) [18], the FibroTest [19], the FAST Fibroscan-Aspartate Aminotransferase Score [20], LSM [21], and the Liver Stiffness Measurement (LSM). Additionally, a number of published studies have explored the effectiveness of Machine Learning (ML) approaches in predicting the different phenotypes of NAFLD [22,23,24,25]. These studies have used various datasets derived from simple blood tests or multi-omics analyses, encompassing logistic regression, random forests, and the XGBoost algorithm for data analysis [26].

The aim of the present study was to characterize the lipidomic profiles associated with the increasing severity of NAFLD and the presence of NASH in patients. The differentiated lipids signatures in the plasma of 15 controls and 22 patients with NAFL and NASH, categorized via liver biopsy, were investigated to identify lipids that may be associated with the disease. Using this information, we explored the application of an ML model for the prediction of NASH, NAFL, or healthy individuals with high accuracy based on specific plasma lipid species.

## 2. Results

### 2.1. Anthropometric and Clinical Characteristics of Study Population

This case-control study comprised 37 individuals, including patients suspected of NAFLD and controls. According to current clinical practice guidelines, the patients were submitted to a percutaneous ultrasound-guided plugged liver biopsy and were categorized as NAFL (27.0%) and NASH (32.5%), based on the NAFLD Activity Score (NAS) evaluation, while the control group comprised 40.5% of the participants, as described by Kalopitas et al. [27]. The demographic and clinical characteristics of the three study groups are presented in Table 1. Parameters such as BMI, MetS, HOMA-IR, and waist circumference were found to differ significantly between the studied groups. In addition, the biochemical parameters ALT, AST, GGT, insulin, HDL-c, total triglycerides, ferritin, uric acid, albumin, HbA1c (%), and NAFLD Activity Score (NAS) were found to differ between the control and the NASH group. ANCOVA analysis was performed to adjust the waist circumference between the groups at the continuous variables, and the adjusted *p*-values are presented in Table 1. After the adjustment, HOMA-IR, the hepatic enzymes ALT, AST, and GGT, insulin, and triglycerides exhibited statistically significant differences between the control and NASH patients.

### 2.2. Investigation of Plasma Lipids Profile in Patients with NAFLD and Controls

**Figure 1:**
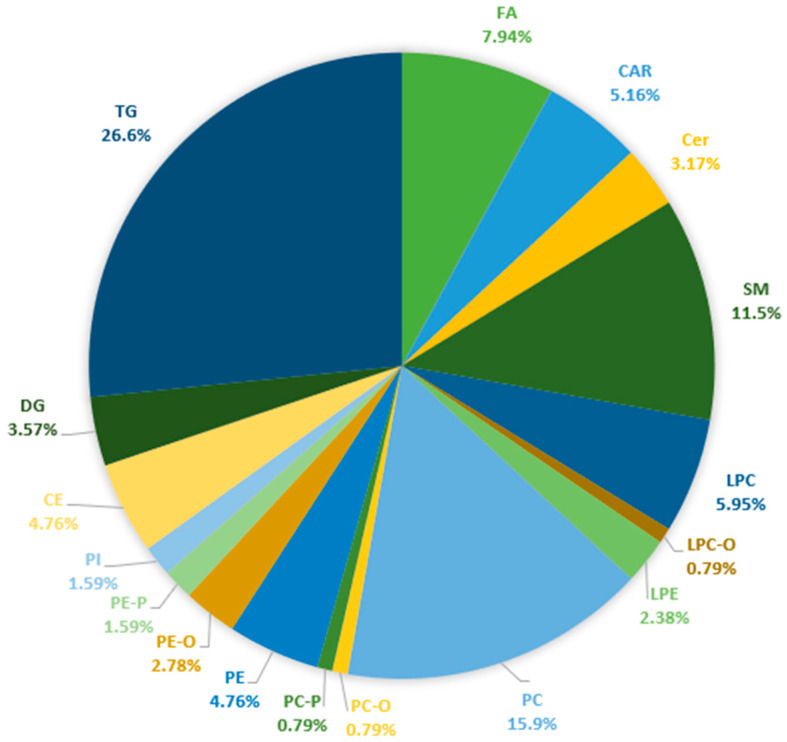
Lipid subclasses quantified in plasma of NAFLD patients based on targeted and untargeted analyses of acylcarnitines, ceramides, fatty acids, and esterified lipids. Abbreviations: FA: Fatty Acids, CAR: Carnitines, Cer: Ceramides, SM: Sphingomyelins, LPC: Monoacylglycerophosphocholines, LPC-O: Monoalkylglycerophosphocholines, LPE: Monoacylglycerophosphoethanolamines, PC: Diacylglycerophosphocholines, PC-O: 1-alkyl,2-acylglycerophosphocholines, PC-P: 1-alkyl,2-acylglycerophosphocholines, PE: Diacylglycerophosphoethanolamines, PE-O: 1-alkyl,2-acylglycerophosphoethanolamines, PE-P: 1-(1Z-alkenyl),2-acylglycerophosphoethanolamines, PI: Diacylglycerophosphoinositols, CE: Cholesterol Esters, DG: Diglycerides, and TG: Triglycerides.

The applied lipidomic workflow facilitated the identification of 359 lipid species, and 215 of them were quantified. Overall, fatty acyls constituted 13.1%, glycerophospholipids 37.3%, glycerolipids 34.9%, and sphingolipids 14.7% of the identified lipid species in blood plasma. Figure 1 illustrates the subclasses of lipids species quantified in the plasma of patients with NAFLD and healthy controls. For a comprehensive overview, the detailed table, including the annotations of molecular species, the molecular formulas, the monoisotopic masses, and the retention time data of all the identified lipids species, is available in Table S1. The validity of the analytical data has been assessed by the analysis of the quality control (QC) samples. The PCA score plots projecting all samples and QC samples provide an indication of satisfactory analytical precision (shown in Figure S1).

**Figure 2:**
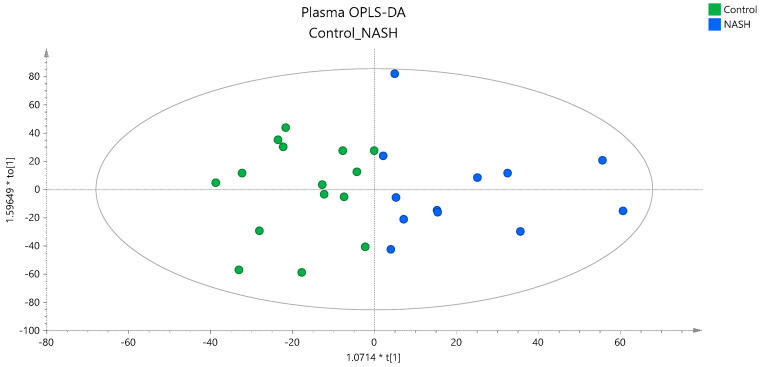
OPLS−DA score plot showing the classification of NASH and controls based on the plasma lipidome.

To investigate whether there is a distinct lipidomic signature associated with the severity of NAFLD, a multivariate statistical analysis was performed based on the quantified lipid species. The unsupervised PCA models constructed could not clearly classify the three groups (data shown in Figure S1). However, the separation of the NASH patients from the other 2 groups could be achieved by partial least squares regression (PLS) analysis, as is presented in the score plot provided in Figure S2. A pairwise OPLS-DA analysis between the studied groups also revealed the differentiation of NASH patients from controls, as is illustrated in the OPLS-DA score plot in Figure 2. Characteristics of the constructed unsupervised and supervised models and validation parameters are provided in Table S2.

Based on multivariate and univariate analyses, nine lipids were identified to contribute to this classification. These statistically significant lipid species are provided in Table 2 along with the estimated *p*-values < 0.05, VIP scores, Log2FC, CV%, median concentrations, and their lower and upper bounds of the 95% confidence intervals (CI). The adjusted *p*-values, based on ANCOVA analysis that was performed for the waist circumference, are also included in Table 2. Three diglyceride species, namely, DG 16:1_18:0, DG 18:0_18:1, DG 18:1_18:1, three phosphatidylcholines PC 16:0_16:1, and PC 18:0_18:1, PC 18:0_22:5, and two phosphatidylinositols, PI 16:0_20:4 and PI 16:1_18:1, and the cholesterol ester CE 20:4, demonstrated a significant impact in the discrimination of the two groups, as all of them, with the exception of CE 20:4, were found to be elevated in the blood plasma of NASH patients. Regarding the discrimination of NAFL patients from controls or NASH patients, no valid model could be constructed based on the blood lipid profiles (data shown in Figure S1).

Data obtained by untargeted and targeted analyses were processed together, considering both the lipidomic data and the clinical and biochemical information of the patients. Therefore, the classification observed between NASH patients and controls through multivariate analysis was further evaluated using an ML approach. This approach considered not only the lipids but also various biochemical parameters to enhance the predictive ability of the data.

### 2.3. Machine Learning Analysis for NAFLD Patients’ Classification

To explore the capability of the extensive information derived from the lipidomics data in distinguishing NAFLD patients from controls, an ML approach was utilized. Various predictive ML models, using the XGBoost algorithm, were created and assessed with the objective of categorizing the participants into controls, NAFL, and NASH patients. The one-vs-rest (OvR) multiclass classification strategy was employed, where each specific group was tested against all the other groups, including the comparisons of controls vs. NAFL–NASH, NAFL vs. controls–NASH, and NASH vs. controls–NAFL.

**Figure 3:**
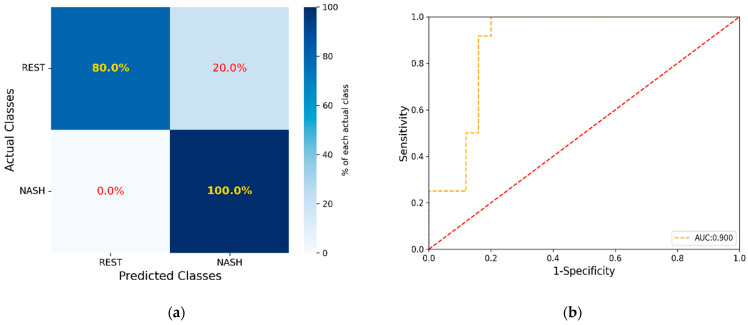
(**a**) Confusion matrix with the results of the sample separation achieved by the model, (**b**) corresponding ROC AUC plot illustration.

**Figure 4:**
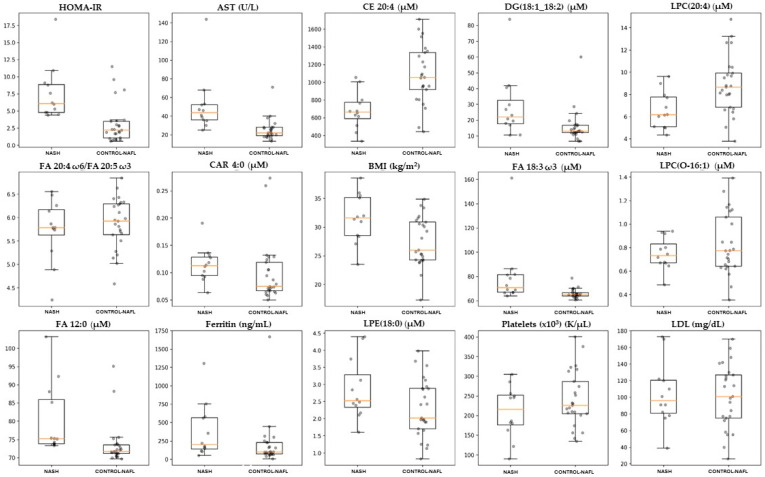
Boxplots illustrating the distribution of the significant lipids and biochemical parameters in blood plasma, identified by the XGBoost algorithm as predictors for NASH patients.

In the initial models for all comparisons, only the data from the lipidomic analysis were included, referred to as the unadjusted models. Among these comparisons, only the NASH vs. controls and NAFL gave satisfying values of the evaluation metrics (MCC: 0.403 ROC AUC score: 0.675 (0.671–0.679 CI 95%), cutoff value: 0.659, accuracy: 70.3%, sensitivity: 75.0%, specificity: 68.0%, PPV: 52.9%, and NPV: 85.0%). The subsequent step involved adjusting the model by incorporating values of biochemical markers, namely, ΒΜΙ, waist circumference, NFS, FIB-4, HOMA-IR, hepatic enzymes ALT, AST, GGT, ALP, platelets, HbA1c %, total cholesterol, total triglycerides, LDL-c, HDL-c, ferritin, and the data obtained from targeted methods, including 13 acylcarnitines, 4 ceramides, 20 fatty acids, and their ratios [27], attaining the optimal performance for the model (MCC: 0.721, ROC AUC score: 0.837 (0.834–0.841 CI 95%), cutoff value: 0.625, accuracy: 86.4%, sensitivity: 91.6%, specificity: 84.0%, PPV: 73.3%, and NPV: 95.5%). The XGBoost algorithm successfully ranked the lipids and the parameters in the dataset according to their importance in achieving discrimination. When the greedy algorithm was applied to evaluate the impact of the number of features on the overall model’s performance (reference model), optimal results were achieved by utilizing the initial 15 most significant lipids and parameters namely, HOMA-IR, BMI, platelet count, LDL-c, ferritin, AST, FA 12:0, FA 18:3 ω3, FA 20:4/FA 20:5, CAR 4:0, LPC 20:4, LPC O-16:1, LPE 18:0, DG 18:1_18:2, and CE 20:4. The outcomes included the following: MCC of 0.721, a ROC AUC score of 0.900 (0.897–0.901 CI 95%), cutoff value: 0.422, accuracy: 86.5%, sensitivity: 100%, specificity: 80.0%, PPV: 70.6%, and NPV: 100%. Figure 3a illustrates the confusion matrix, depicting the outcomes of sample separation achieved by the model. In Figure 3b, the corresponding ROC AUC plot is presented. The model successfully classified 20 out of 25 individuals (80.0%) in the control and NAFL group and accurately identified 12 out of 12 patients (100%) in the NASH group. Table 3 provides a summary of the biochemical parameters and lipids from the dataset involved in the optimized model. The concentration distribution of lipids and biochemical parameters between the two groups are also depicted in Figure 4. The optimal model was validated using the permutation test, of which results are provided in Figure S3.

## 3. Discussion

In this study, we explored the results obtained from a lipidomic-based examination of plasma samples, collected from 37 controls and patients with suspicion of NAFLD, classified into NAFL or NASH based on liver histology. Our analysis focused on variations in lipidomes, specifically in fatty acids, acylcarnitines, ceramides, sphingomyelins, phosphatidylinositols, (Lyso)phosphatidylethanolamines, (Lyso)phosphatidylcholines, diglycerides, triglycerides, and cholesterol esters, aiming to identify lipids that could be linked to the phenotype and complexity of NAFLD. Untargeted and targeted approaches in lipids analysis were combined with biochemical markers from a simple blood test and baseline characteristics providing a panel of lipids and biochemical parameters which was used to develop an ML predictive algorithm able to evaluate the stratification of the disease. The one-vs-rest (OvR) multiclass classification strategy was utilized, exhibiting satisfactory values for the evaluation metrics MCC and AUC ROC only for the NASH vs. controls and NAFL comparison. According to our final ML model, accurate prediction of NASH patients was attained utilizing lipidomic data, markers from a simple biochemical test, and baseline characteristics, incorporating 15 features into the model.

Using ML to predict diseases enables broader identification, timely intervention, and precise treatments to enhance or manage disease progression. Previous research has also explored the effectiveness of ML in predicting or diagnosing different stages of NAFLD. The primary reference for these studies is a recent publication by Nourenddin et al., who developed the metabolomics-advanced steatohepatitis fibrosis score (MASEF). This algorithm utilized a multivariate logistic regression model that incorporated 12 lipids (2 triglycerides, 5 glycerophosphatidylcholines, 1 cholesterol ester, 1 ceramide, and 3 sphingomyelins), BMI, ALT, and AST enzymes, achieving the highest AUC in the validation cohort of 565 patients with high-risk metabolic dysfunction-associated steatohepatitis (MASH). The MASEF score characteristics included an AUC of 0.789 (0.750–0.827, 95% CI), cutoff of 0.33, accuracy of 69.0%, sensitivity of 78.2%, specificity of 65.2%, PPV of 48.1%, and NPV of 87.9% [23]. In addition to this study, Atabaki-Pasdar et al. employed a least absolute shrinkage and selection operator (LASSO) model for feature selection to develop a series of random forest models and predict whether liver fat content was <5% or ≥5% in a population of 1514 non-NAFLD and NAFLD individuals, respectively, using a combination of multi-omics and clinical variables as predictors. Their optimal model achieved an AUC score of 0.82, accuracy of 74.0%, sensitivity of 74.0%, and specificity of 73.0% using nine clinically available features [22]. In the study by Perakakis et al., an ML model for NASH prediction was also devised, including 31 NASH/NAFL patients and 49 healthy individuals, where a one-vs-rest (OvR) approach was employed. In total, they measured 365 lipid species in addition to glycans and hormones. The optimal models included either 29 lipid species or a total of 20 features, incorporating lipids, glycans, or hormones. Although the authors achieved high performance with an AUC score of 0.95, accuracy of 88.0%, sensitivity of 89.0%, and specificity of 94.0%, the extensive array of laboratory markers they utilized is not typically included in routine clinical care, thus, this model would likely necessitate specific additional testing and could not be easily applied to existing Electronic Health Record (EHR) data [24]. The primary goal of the study of Yaghouti et al. was to create an ML model that utilizes clinical data and blood parameters to predict NASH using the NAS in 181 patients. Among the various classifiers explored, the random forest model, in combination with Sequential Feature Selection (SFS), demonstrated the optimal performance, with an accuracy of 81.3%, sensitivity of 86.0%, and specificity of 70.5% [25]. In our approach, the XGBoost algorithm, which is widely used in ML approaches, was employed, utilizing gradient boosting with decision trees as the underlying learners. In contrast to random forests, where individual trees work independently to address the problem, XGBoost constructs its trees sequentially. Each tree is trained to mitigate the prediction error left by the preceding tree, thereby enhancing the prediction accuracy. This approach offers an alternative method for constructing more sophisticated and precise models using trees while managing the depth and complexity of each individual tree. Additionally, XGBoost has exhibited robust performance in various studies related to NAFLD that employed an ML approach [28,29]. Based on our results, we achieved accurate prediction results for NASH patients against controls-NAFL groups, including an MCC of 0.721, a ROC AUC score of 0.900 (0.897–0.901 CI 95%), cutoff value: 0.422, accuracy: 86.5%, sensitivity: 100%, specificity: 80.0%, PPV: 70.6%, and NPV: 100%. These predictions were achieved by utilizing lipidomic data, markers from a simple biochemical test, and baseline characteristics, namely, HOMA-IR, BMI, platelets, LDL-c, ferritin, AST, FA 12:0, FA 18:3 ω3, FA 20:4/FA 20:5, CAR 4:0, LPC 20:4, LPC O-16:1, LPE 18:0, DG 18:1_18:2, and CE 20:4.

Some of the aforementioned biochemical markers are widely acknowledged as risk factors closely associated with NAFLD, such as HOMA-IR and BMI. In our study, elevated HOMA-IR values were observed in the NASH group, indicating hepatic and adipose tissue IR attributing to NAFLD progression [30,31]. The role of platelets in the progression of NASH has been recognized. The higher concentration of platelets observed in the liver of NASH patients is related to both NAS and the formation of intrahepatic NETs. The interaction between platelets and neutrophils has been identified as a key factor in NET-induced thromboinflammation. A recent study by Arelaki et al. found a negative association between NETs and platelets in liver biopsies, which may explain the low peripheral platelet counts observed in some patients with early stages of NASH, which aligns with our findings [32]. Ferritin constitutes the primary storage protein for iron in the liver [33] and it is also identified as an acute phase protein that can be triggered in response to systemic inflammation [34]. In our study, ferritin levels were found to be elevated, suggesting that an increase in iron deposition plays a crucial role in initiating the production of reactive oxygen species through the Fenton reaction, which may lead to liver inflammation, elevated oxidative stress, and ultimately contribute to steatohepatitis and fibrosis [35].

Recent metabolomic and lipidomic studies have demonstrated that NAFLD is accompanied by disturbed levels of diacylglycerols (DGs), free cholesterol, phosphatidylcholines (PCs), and altered metabolism of saturated fatty acids (SFAs), monounsaturated fatty acids (MUFAs), and polyunsaturated fatty acids (PUFAs), including ω3 and ω6 fatty acids [13]. Our results revealed that FA 12:0 is more prevalent in patients with NASH, indicating a possible link between NASH and the accumulation of saturated fatty acids in hepatocytes, leading to oxidative stress and inflammasome activation resulting in cell damage and apoptosis [27]. PUFAs have various biological functions, including proinflammatory and anti-inflammatory properties, highlighting a possible relation in the development of NASH. In our study, FA 18:3 ω3 was significantly higher in NASH patients, indicating increased lipid oxidation and PPARα activation, which results in enhanced energy expenditure. This finding exhibits an association with the results from Kalhan et al., where they found significantly higher levels of FA 18:3 ω3 in the steatosis group in comparison with NASH patients [36,37]. Furthermore, FA 20:4 ω6 (AA) and FA 20:5 ω3 (EPA) are precursors to important inflammatory mediators and their ratio can provide insight into inflammation and nutritional status of cell membranes. An imbalanced AA/EPA ratio in favor of AA has been linked to the development of various metabolic disorders, including obesity, cardiovascular disease, and NAFLD. Although the exact mechanism underlying the connection between the AA/EPA ratio and NAFLD is not yet clear, a study by Tutino et al. suggested that the inflammatory effects of AA contribute to liver injury [38]. In our study, the AA/EPA ratio was identified as a significant factor for group classification by the algorithm; however, the ratio was found in similar levels between the compared groups. The analysis of existing data highlighted significant discrepancies regarding the concentrations of PUFAs and their association with the progression of NAFLD across multiple studies [39,40,41]. The XGBoost algorithm identified CAR 4:0 as a significant compound for the classification of NASH patients and control–NAFL groups. In particular, acylcarnitine species in serum have been associated with the promotion and secretion of inflammatory cytokines from immune cells in individuals with NAFLD [42,43].

Phospholipids are essential for the structure and function of the plasma membrane, for very low-density lipoprotein (VLDL), and for signaling pathways like PI3K. When PC levels are reduced, the liver secretes less VLDL cholesterol, leading to lipid accumulation in hepatic cells [44] and an imbalance in the ratio of hepatic PC to PE [45]. In our study, PC 16:0_16:1 was found to be elevated in the NASH group, indicating the association between the composition of fatty acids and circulating PCs. Puri et al. [46] investigated the composition of fatty acids in plasma PCs, revealing that the levels of palmitoleic acid were increased in PCs in both NAFL and NASH patients, whereas palmitic acid levels did not differ significantly compared to normal. This observation was also confirmed by another study, where circulating PCs were increased in NAFL and NASH patients compared to healthy controls [47]. Regarding LPC species, LPC 20:4 levels were significantly decreased in NASH patients, while LPC O-16:1 concentration remained at similar levels across the study groups. These lipids are abundant in HDL-c particles and their decreased level in NASH patients may reflect the known negative association between HDL-c and liver fat content [48]. The XGBoost algorithm identified LPE 18:0 as a phospholipid with the ability to discriminate NASH and control–NAFL groups in our study. In a relative publication, LPE levels were found to be higher in a steatosis group compared to in normal liver tissue, but not in NASH, suggesting increased lipid turnover rather than hydrolysis [11]. Two PI species, namely, PI 16:0_20:4 and PI 16:1_18:1, were found to be elevated in the NASH group of our study. A similar finding was observed by Ma et al., who revealed that patients with steatosis and NASH had higher plasma concentrations of PI compared to healthy controls. PI and its related metabolites serve as crucial second messengers that participate in the signaling pathways of mitogen-activated protein kinase and protein kinase B (PKB/Akt) [49].

Certain lipid mediators, including neutral lipids, are often associated with lipotoxicity and are considered to play a crucial role in the progression of NASH. Our investigation revealed that DGs and CEs were notable factors in distinguishing NASH patients from the control–NAFL group. Specifically, NASH patients exhibited elevated concentrations of DGs in their plasma, while CE 20:4 levels were reduced in this group. Prior research by Gorden et al. indicated that DGs were less abundant in NASH compared to the steatosis group, and slightly lower in NASH than in normal individuals [11]. The DG trends in NASH reflected those observed in cirrhosis, and several CE species also displayed lower levels in NASH compared to steatosis. In a relative study, the fatty acids derived from DG species contained palmitic, stearic, and oleic acids in controls, NAFL, and NASH patients. Notably, palmitoleic acid levels significantly increased in both NAFL and NASH within DGs. Furthermore, there was a noteworthy decrease in stearic acid (FA 18:0) levels and a corresponding rise in its downstream product, oleic acid (FA 18:1 n9), in DG in both NAFL and NASH groups [46].

Nevertheless, this study is limited by the relatively modest sample size and the absence of data pertaining to participants’ dietary habits. The optimal combination of features might differ depending on the specific population under investigation, given that NASH prevalence varies among different ethnicities. Enhancing the model by training it on a more extensive dataset that incorporates diverse ethnic groups could provide valuable insights into the pathophysiological mechanisms associated with NASH onset and progression. Additionally, refining the model to predict the exact NAS and fibrosis stage would be a significant advancement. However, validating the diagnostic potential of this biomarker panel requires a rigorous process involving a substantial and diverse participant cohort across multiple centers. Moreover, practical implementation in clinical practice will necessitate considerations related to repeatability and cost-effectiveness.

## 4. Materials and Methods

### 4.1. Study Population

The current study was a case-control trial, comprised of three different groups of subjects: individuals with verified NAFLD, including both NAFL and NASH, as determined by biopsy results, and a control group, consisting of healthy individuals. The criteria of the study are described in detail in an earlier publication of our group [27]. The initial diagnosis of NAFLD was conducted radiologically and the absence of fatty liver disease was determined in healthy controls by normal values of MRI-PDFF (Magnetic Resonance Imaging-Proton Density Fat Fraction), normal liver biochemistry, and the absence of other chronic liver diseases. Patients with NAFLD were further stratified into two subgroups, NAFL and NASH, based on their liver biopsies. All participants provided written informed consent and were recruited into the study between June 2021 and June 2023. The research adhered to the principles outlined in the Declaration of Helsinki [50], received approval from the Institutional Review Board of the Medical School of Aristotle University of Thessaloniki, and underwent scrutiny and approval by the Bioethics Board of the Medical School of Aristotle University of Thessaloniki, with the assigned protocol number being 4.399/26/01/2021. Blood was collected from all individuals for lipid analysis after an overnight fast and a homogeneous low-fat diet for the past 24 h. Blood was centrifuged, plasma was separated and immediately stored at −80 °C until the analysis. In this study, the patients met both the old and the new nomenclature criteria.

### 4.2. Chemicals and Materials

Methanol (MeOH), acetonitrile (ACN), methyl-tert-butyl-ether (MTBE; ≥99%), and formic acid (all ULC/MS-CC/SFC grade) were obtained from CHEM-LAB NV (Zedelgem, Belgium). Isopropanol (IPA) was purchased from Fisher Scientific (International Inc., Hampton, NH, USA). Ammonium formate (NH_4_HCO_2_; MS grade) and 2,6-di-tert-butyl-4-methylphenol (BHT) were obtained from Sigma-Aldrich (Merck, Darmstadt, Germany). Deionized water (ddH_2_O) was ultra-purified by a Millipore (Bedford, MA, USA) instrument delivering water quality of a resistivity ≥ 18.2 MΩ∙cm. SPLASH^®^ LIPIDOMIX^®^ was purchased from Avanti Polar Lipids (Avanti Polar Lipids, Inc., Alabaster, AL, USA).

### 4.3. Acylcarnitines, Ceramides, and Fatty Acids Analyses in Plasma

Analyses of acylcarnitines, ceramides, and fatty acids in plasma were performed using methods developed by our group. Briefly, for carnitine analysis, a HILIC-MS/MS method was used for the quantitation of 13 acylcarnitines [51]. Four (4) ceramides species, namely, Cer(d18:1/16:0), Cer(d18:1/18:0), Cer(d18:1/24:0), and Cer(d18:1/24:1) were determined by UHPLC-MS/MS [52]. Fatty acids were esterified to their methyl esters and analyzed using the GC-MS method [53]. In total, 20 fatty acid methyl esters were quantified.

### 4.4. Extraction Protocol for Plasma Lipidomics Analysis

For the UHPLC-TOF-MS/MS lipidomics analysis, 50 µL of plasma samples were thawed on ice for 30 min. Five (5) µL of SPLASH^®^ LIPIDOMIX^®^ were added to each sample with subsequent incubation on ice for 15 min. For lipid extraction, 375 µL MeOH and 1250 µL of MTBE were added, followed by vortexing. Samples were shaken for 30 min at room temperature. Phase separation was enhanced by adding 375 µL of H_2_O and the samples were shaken for another 10 min at room temperature. After the end of incubation, samples were centrifuged for 10 min at 4 °C and 10,000 rpm. The organic phase was collected, transferred into 2 mL Eppendorf tubes, and evaporated to dryness under vacuum (SpeedVac, Eppendorf Austria GmbH, Wien, Austria). The dried samples were reconstituted with IPA (200 μL for negative ionization mode and 400 μL for positive ionization mode). The injection volume for positive ionization was 3 μL, while for negative mode the injection volume was 10 μL. A Quality Control sample (QC) was prepared as representative by mixing equal volumes of each serum sample. Group-specific QC samples for control, NAFL, and NASH were prepared as well. Diluted QCs (1:2, 1:4, 1:8) in IPA were also analyzed to evaluate the dilution integrity of the detected lipids. All solvents contained 0.01% (*w*/*v*) BHT and were cooled on ice before use.

### 4.5. Instrumentation

An UHPLC Elute system equipped with an Elute autosampler was used. The autosampler vial tray was maintained at 8 °C whereas the needle was washed with 2500 μL of a strong wash solvent (IPA/ACN/MeOH/H_2_O at a ratio of 30/30/30/10) and 1000 μL of a weak wash solvent (ACN/H_2_O, 60/40) before and after each injection. A 30 min gradient elution was employed using a binary solvent manager. The mobile phase A consisted of ACN/H_2_O (50:50) and mobile phase B of IPA/ACN/H_2_O (85:10:5), both containing 5 mM ammonium formate and 0.1% formic acid (FA). The gradient profile was as follows: 0–20 min—10 to 86% B, 20–22 min—86 to 95% B, 22–26 min –95% B isocratic, 26–26.1 min—95 to 10% B, 26.1–34.0 min—10% B isocratic, and column re-equilibration. The flow rate was set at 0.3 mL/min. An Acquity UPLC CSH C18, 2.1 × 100 mm, 1.7 μm column (Waters Ltd., Elstree, UK) equipped with a pre-column Acquity UPLC CSH C18 Van-Guard (Waters Ltd., Elstree, UK) was used for chromatographic separation and maintained at 50 °C.

A TIMS TOF mass spectrometer (Bruker, Billerica, MA, USA) was used in both positive and negative ionization modes for MS and MS/MS data acquisition. Data-dependent acquisition (DDA) was performed to enhance the annotation of lipids. The parameters at source were set as follows: end plate offset was set to 500 V and the capillary voltage was set at ±4500 V for positive and negative modes, respectively. Nitrogen was used as the dry gas at the rate of 10 L/min and dry temperature of 200 °C. The nebulizer gas was set at 2.0 bar. The peak detection threshold was set at 100 counts. In DDA analysis, auto MS/MS was applied for the 10 most intense ions per scan using Dynamic MS/MS spectra acquisition with 6 and 10 Hz as minimum and maximum spectra rates, respectively. Collision energy was set at 20 V for precursor ions below 100 *m/z*, 30 V for precursor ions with *m/z* ranging from 100 to 1000, and 40 V for precursor ions with *m/z* ranging from 1000 to 2000 *m/z*. Calibrant (sodium formate, 10 mM) was infused into MS during 0.1–0.3 min with a flow rate of 10.0 µL/h.

### 4.6. Identification and Qualification of Lipids Species

Identification of lipid species was performed in Lipostar2 (version 2.0.2 Molecular Discovery Ltd., Hertfordshire, UK) equipped with the LIPID MAPS structure database (version September 2021) [54]. The raw data files from the QC and group-specific QC samples acquired in positive and negative ionization modes were imported directly into the software and aligned using the default settings. Automatic peak picking was performed with the Savitzky–Golay algorithm using the following parameters: window size set to 7, degree to 2, multi-pass iterations to 1, and minimum S/N ratio to 3. Mass tolerance settings were set to 10 ppm with an RT tolerance of 0.2 min. The filters “Retain lipids with isotopic pattern” and “Retain lipids with MS/MS” were applied to keep only features with isotopic patterns and MS/MS spectra for identification. The following parameters were used for lipid identification: 5 ppm precursor ion mass tolerance and 20 ppm product ion mass tolerance. The automatic approval was performed to keep structures with a quality of 3–4 stars.

To confirm the accuracy of lipid annotations, the retention time of given lipid species against their Kendrick mass defect to the hydrogen base was plotted using an in-house script in the Python programming language. For a comprehensive understanding of how retention time mapping was conducted for various lipid (sub)classes, a more detailed description is referenced by Lange et al. [55].

For the quantification of sphingolipids, phospholipids, and glycerolipids, Skyline v.21.1.0.146 (MacCoss Lab) [56] was employed. Only data from the positive mode were used, and each lipid underwent quantification by selecting the corresponding precursor ion for peak integration. The peak boundaries were defined, manually adjusted, and verified. To normalize the obtained peak areas, specific lipid species from the SPLASH^®^ Lipidomix Mass Spec Standard (Avanti) were utilized: d18:1-18:1(d9) SM for ceramides and sphingomyelins, 18:1(d7) Lyso PC for lysophosphatidylcholines, 18:1(d7) Lyso PE for lysophosphatidylethanolamines, 15:0-18:1(d7) PC for phosphatidylcholines, 15:0-18:1(d7) PE phosphatidylethanolamines, 15:0-18:1(d7) DAG for diglycerides and 15:0-18:1(d7)-15:0 TAG for triglycerides.

Type I isotopic correction and correction for the incomplete labeling of deuterated internal standards (ISTDs) were applied. The quantitative values for lipid species were determined by dividing the corrected peak area for each lipid species by the peak area of the respective ISTD and then multiplying it by the concentration of the specific ISTD for each lipid class.

### 4.7. Data Analysis and Visualization

Univariate statistical analysis was conducted in the Python programming language and GraphPad Prism v8.0.1 software. Continuous data in baseline characteristics are presented as median (25th–75th percentile), while categorical data are expressed as counts and percentages. The distributions were evaluated using the Shapiro–Wilk test. A one-way ANOVA was conducted for normally distributed features followed by Bonferroni adjustment, while the Kruskal–Wallis test was used for non-normally distributed features. Post hoc Dunn’s test was applied when the *p*-value was less than 0.05. The chi-square test was utilized in categorical parameters. Statistical significance was defined as *p*-value < 0.05, and the differentiated lipids are presented as median concentrations with lower and upper bounds of the 95% confidence intervals (CI). Following this, ANCOVA analysis was conducted to account for waist circumference differences between groups, a significant parameter affecting intra-abdominal fat. This adjustment aimed to ensure that the observed differences between the groups could be attributed to the varying stages of disease. Non-normally distributed features were logarithmically transformed before the ANCOVA analysis. An adjusted *p*-value < 0.05 was considered significant. Only lipids with a *p*-value <0.05 before and after the ANCOVA analysis were considered statistically significant. To assess the statistical significance of variables derived from the algorithm, a comprehensive statistical analysis was conducted. Student’s *t*-test was applied to parameters demonstrating a normal distribution, while the Mann–Whitney U test was employed for variables exhibiting non-normal distribution characteristics, as both the control and NAFL groups were combined and compared against NASH patients for the analysis.

Multivariate statistical analysis was carried out using SIMCA 13.0.3 (UMETRICS AB Sweden) [57], and the data were processed using an unsupervised principal component analysis (PCA), partial least squares analysis (PLS), and orthogonal-partial least squares discriminant analysis (OPLS-DA). The identification of significant lipids was performed using an “S-plot” with absolute p and p (corr) values cut off. Features meeting the criteria of *p* > |0.05| and *p* (corr) > |0.5| were considered statistically significant. To assess the model quality, parameters such as the goodness of fit in the X (R^2^X) and Y (R^2^Y) variables, as well as predictability (Q^2^), were assessed through the software. A *p*-value from the CV ANOVA analysis indicating the statistical significance of the model was calculated using the software as well. Logarithmic transformation of the data and pareto scaling were used in all models.

A machine learning (ML) approach was employed, using the XGBoost algorithm [58] with the aim of exploring the potential of differentiated lipid species to predict distinct disease stages. ML predictive models were generated using a double cross-validation (nested) approach, where the F1 score was used as the optimization metric [59]. The optimal 1-score cutoff was determined as the point where Youden’s J statistic (or J point) was maximized, corresponding to the point where sensitivity + (1-specificity) was maximum. Matthews Correlation Coefficient (MCC) and receiver operating characteristic area under the curve (ROC AUC) scores were used as the models’ performance metrics and were evaluated along with accuracy, sensitivity, specificity, positive predictive value (PPV), and negative predictive value (NPV). The AUC is reported, along with a 95% CI calculated with 1500 stratified bootstrap replicates. Each model underwent evaluation five times, employing distinct randomization settings in each iteration to gauge the robustness of the results. A one-vs-rest (OvR) multiclass classification strategy was used, testing each individual group against all other groups (control vs. NAFL–NASH, NAFL vs. control–NASH, and NASH vs. control–NAFL) [60]. Subsequently, a greedy algorithm was employed to identify the optimal combination of the most significant features in the dataset, aiming for the optimal prediction results. Initially, the prediction model was trained using the complete set of features in the dataset. Thereafter, the features were sorted in descending order, considering the significance coefficient assigned to them by the XGBoost algorithm. Various subsets of the original dataset were produced, progressively integrating an increased number of the most significant features. These subsets ranged from 1 to 25 features and were ultimately assessed for the predictive performance of the models they produced. The optimal predictive model was then validated using the permutation test as described by Lindgren et al. [61]. A collection of 200 permuted response variables was generated by randomly rearranging the entry values of the original response variable. These permuted response variables were individually utilized to construct the corresponding prediction models. During each iteration, the values of evaluation metrics were computed and recorded. Finally, the outcomes of the permuted models were compared with those of the reference model, which was developed using the intact response variable. All data and statistical analyses were performed using in-house scripts developed in the Python programming language.

## 5. Conclusions

The high-dimensional nature of lipidomic data, coupled with the multitude of lipids and clinical markers, often require advanced ML approaches to unravel the intricate lipidomic interactions, utilizing a large pool of biomarkers for risk-stratification and shedding light on their role in NAFLD. Our research findings reveal that patients with NASH exhibit a unique plasma lipid profile, which differentiates them from NAFL patients and controls. This plasma lipid profile appears to align with the level of histological activity, suggesting that plasma lipids could serve as a beneficial biomarker for identifying NASH. The novelty of this study is demonstrated by the precision of the obtained results and the powerful diagnostic performance of the generated model, which contribute to the existing body of evidence and emphasize the need for further investigation in this area. However, further validation of our results is needed in larger patient populations.

## Figures and Tables

**Table 1:** Baseline characteristics of the study population and comparison between control, NAFL, and NASH groups.

Parameters	Total Population	Control	NAFL	NASH	*p*-Value	Adjusted *p*-Value with Waist Circumference
Demographics and clinical characteristics						
Sex (Male)	37 (22)	15 (8)	10 (6)	12 (8)	7.81 × 10^−1^	7.81 × 10^−1^
Age (years)	54.0 (46.0–60.0)	53.0 (41.5–55.0)	57.0 (53.2–60.0)	57.5 (45.8–65.5)	3.05 × 10^−1^	8.76 × 10^−1^
BMI (kg/m^2^)	29.3 (24.9–31.8)	25.3 (24.0–28.7)	31.1 (26.6–33.0)	31.6 (28.6–35.2)	1.70 × 10^−3 a,b^	5.11 × 10^−1^
Diabetes Mellitus	13 (35.1%)	3 (20%)	3 (30%)	7 (58.3%)	1.08 × 10^−1^	1.08 × 10^−1^
Arterial Hypertension	12 (32.4%)	3 (20%)	4 (40%)	5 (41.7%)	4.09 × 10^−1^	4.09 × 10^−1^
Metabolic Syndrome	16 (43.2%)	3 (18.7%)	4 (25%)	9 (56.3%)	1.60 × 10^−2 a,b^	1.60 × 10^−2 a,b^
Waist Circumference (cm)	100 (95.0–112)	95.0 (83.5–98.0)	106 (97.0–118)	111 (100–120)	2.00 × 10^−3 a,b^	3.62 × 10^−3 a,b^
HOMA-IR	3.50 (1.90–6.20)	1.90 (1.05–2.55)	3.30 (1.92–8.00)	6.10 (4.80–8.88)	1.66 × 10^−4 b^	5.02 × 10^−3 b^
Biochemical parameters						
NAS	3 (1.75–5)	0	2.5 (2–3)	5 (4–6)	6.16 × 10^−6 b^	6.16 × 10^−6 b^
ALT (U/L)	28.0 (22.0–51.0)	20.0 (15.5–26.0)	35.5 (23.2–50.5)	48.5 (39.0–92.5)	3.20 × 10^−4 b^	9.94 × 10^−5 b^
AST (U/L)	27.0 (20.0–39.0)	20.0 (18.0–23.5)	27.0 (23.8–31.0)	43.5 (35.8–52.2)	6.44 × 10^−5 b^	1.14 × 10^−4 b^
GGT (U/L)	23.0 (13.0–54.0)	13.0 (11.0–20.0)	25.0 (18.5–56.2)	52.5 (35.0–93.5)	2.23 × 10^−3 b^	4.82 × 10^−2^
ALP (U/L)	72.0 (55.0–93.0)	58.0 (53.5–89.0)	77.5 (62.8–96.8)	86.5 (64.8–94.2)	2.81 × 10^−1^	6.58 × 10^−1^
Insulin (μlU/mL)	12.3 (7.40–25.8)	9.50 (4.40–11.2)	14.4 (7.45–31.6)	25.4 (21.9–27.8)	2.00 × 10^−4 b^	3.53 × 10^−2 b^
Platelets (×10^3^) (K/μL)	226 (182–274)	217 (206–255)	253 (187–314)	215 (175–251)	3.69 × 10^−1^	4.22 × 10^−1^
HbA1c (%)	5.50 (5.20–6.00)	5.40 (5.10–5.70)	5.50 (5.22–5.75)	6.15 (5.72–7.02)	4.67 × 10^−2 b^	1.32 × 10^−1^
FBG (mg/dL)	96.0 (88.0–110)	89.0 (84.5–96.5)	94.5 (85.0–111)	109 (94.8–134)	6.25 × 10^−2^	1.86 × 10^−1^
Total cholesterol (mg/dL)	185 (147–202)	177 (155–200)	192 (125–215)	192 (168–200)	5.66 × 10^−1^	5.83 × 10^−1^
LDL-c (mg/dL)	101 (77.0–126)	99.0 (76.0–128)	114 (60.5–126)	96.0 (81.0–120)	8.61 × 10^−1^	8.32 × 10^−1^
HDL-c (mg/dL)	50.0 (42.0–60.0)	56.0 (53.5–64.0)	45.5 (32.2–54.8)	43.0 (40.8–49.2)	6.30 × 10^−3 b^	5.02 × 10^−2^
Triglycerides (mg/dL)	116 (85.0–178)	84.0 (60.0–100)	128 (95.2–150)	192 (144–283)	2.00 × 10^−4 b^	1.26 × 10^−3 b^
Ferritin (ng/mL)	149 (79.7–249)	97.0 (65.2–160)	136 (84.4–293)	200 (140–564)	2.75 × 10^−2 b^	2.98 × 10^−1^
Uric acid (mg/dL)	4.90 (4.30–5.80)	4.80 (4.00–5.10)	4.85 (4.70–5.92)	5.60 (4.40–6.75)	3.00 × 10^−2 b^	2.48 × 10^−1^
Albumin (gr/dL)	4.58 (4.38–4.70)	4.60 (4.36–4.70)	4.57 (4.51–4.68)	4.50 (4.36–4.62)	1.00 × 10^−2 b^	4.89 × 10^−1^

Continuous variables are presented as median (25th–75th percentile). Categorical parameters are presented as counts and percentages for each parameter’s category. A one-way ANOVA and Kruskal–Wallis tests were conducted for normally and non-normally distributed continuous parameters, respectively, while a Chi-square (χ^2^) test was conducted for the categorical variables, in order to assess the statistical significance of the comparison between the three distinct NAFLD groups. The threshold for statistical significance was set at *p* < 0.05. Abbreviations: BMI Body Mass Index, HDL-c high-density lipoprotein, LDL-c low-density lipoprotein, ALT Alanine transaminase, AST Aspartate Aminotransferase, GGT Gamma-glutamyl Transferase, ALP Alkaline Phosphatase, FBG Fasting Plasma Glucose. Statistically significant parameters between the groups: ^a^ control–NAFL, ^b^ control–NASH.

**Table 2:** Lipids with statistical significance according to the clinical manifestation of NAFLD.

Plasma											
Control–NASH						Control (N = 15)			NASH (N = 12)		
Lipids	*p* Value	*p* Value Adj Waist	VIP	Log2FC	CV%	Median	95% Lower CI	95% Upper CI	Median	95% Lower CI	95% Upper CI
						μΜ			μΜ		
**CE 20:4**	7.00 × 10^−4^	3.20 × 10^−3^	7.4	−0.76	3.89	1093	959	1295	664	564	783
**DG(16:1_18:0)**	6.85 × 10^−4^	3.18 × 10^−3^	1.3	1.68	8.43	4.87	4.13	5.15	9.59	8.22	21.9
**DG(18:0_18:1)**	7.41 × 10^−4^	1.57 × 10^−3^	0.6	1.48	6.17	1.33	1.13	1.51	2.8	2.17	5.79
**DG(18:1_18:1)**	1.67 × 10^−3^	1.15 × 10^−2^	1.6	1.03	8.44	14.9	13.3	17.1	23.0	19.9	43.9
**PC(16:0_16:1)**	1.85 × 10^−2^	2.00 × 10^−2^	1.3	0.78	0.99	17.9	13.2	20.2	26.5	19.8	39.7
**PC(18:0_18:1)**	2.66 × 10^−2^	2.90 × 10^−2^	1.6	0.42	0.75	50.0	44.7	55.4	65.7	50.7	82.6
**PC(18:0_22:5)**	1.35 × 10^−2^	4.49 × 10^−2^	0.7	0.47	0.91	12.2	11.7	13.8	18.7	15.1	20.2
**PI(16:0_20:4)**	7.30 × 10^−3^	1.42 × 10^−2^	0.7	0.91	17.1	4.03	3.17	5.62	6.67	3.89	12.3
**PI(16:1_18:1)**	7.85 × 10^−3^	1.36 × 10^−2^	0.8	1.01	20.8	3.35	3.03	4.81	6.20	5.15	9.56

**Table 3:** Lipids enhanced the classification of NASH patients as derived from the XGBoost algorithm.

		Total Population			NASH			Control–NAFL		
	*p*-Value	Median	95% Lower CI	95% Upper CI	Median	95% Lower CI	95% Upper CI	Median	95% Lower CI	95% Upper CI
**HOMA-IR**	2.61 × 10^−4^	3.50	2.20	4.80	6.10	4.80	8.95	2.2	1.70	2.90
**BMI (kg/m^2^)**	1.04 × 10^−2^	29.3	25.7	31.2	31.6	28.5	35.3	26.0	24.4	30.4
**Platelets** **(×10^3^) (K/μL)**	1.87 × 10^−1^	226	205	251	216	172	253	226	207	274
**LDL** **(mg/dL** **)**	8.10 × 10^−1^	101	82.0	121	96.0	80.0	121	101	77.0	126
**Ferritin** **(ng/mL)**	2.62 × 10^−2^	149	98.7	218	200	132	569	101	76.0	163
**AST** **(U/L)**	8.47 × 10^−5^	27.0	23.0	35.0	43.5	35.5	52.5	22.0	20.0	27.0
**FA 12:0 (μΜ)**	5.50 × 10^−4^	73.4	71.7	74.0	75.2	73.8	86.7	71.7	71.2	73.4
**FA 18:3 ω3 (μΜ)**	1.11 × 10^−3^	66.0	65.2	68.9	70.8	66.9	81.5	65.3	64.7	66.0
**FA 20:4 ω6/** **FA 20:5 ω3**	4.79 × 10^−1^	5.86	5.74	6.14	5.79	5.51	6.20	5.92	5.66	6.27
**CAR 4:0 (μΜ)**	4.18 × 10^−1^	0.09	0.07	0.11	0.11	0.09	0.13	0.07	0.07	0.11
**LPC(20:4) (μΜ)**	9.60 × 10^−3^	7.99	6.71	8.75	6.16	5.07	7.81	8.66	7.99	9.76
**LPC(O-16:1) (μΜ)**	3.25 × 10^−1^	0.74	0.68	0.85	0.73	0.67	0.86	0.77	0.67	1.00
**LPE(18:0) (μΜ)**	7.95 × 10^−2^	2.43	1.97	2.86	2.53	2.28	3.44	2.02	1.90	2.88
**DG(18:1_18:2) (μΜ)**	8.19 × 10^−3^	15.2	12.6	18.0	22.0	17.5	35.2	13.0	12.3	16.5
**CE 20:4 (μΜ)**	4.00 × 10^−4^	957	766	1056	664	564	783	1056	957	1295

## Data Availability

Dataset available on request from the authors.

## Supplementary Materials

The following supporting information can be downloaded at the following location: https://www.mdpi.com/article/10.3390/ijms25115965/s1. Figure S1: PCA score plot for three studied groups and QC samples was constructed. Control group is illustrated in green, NAFL group in purple and NASH group in blue, whereas QC samples are depicted in grey and clustered together. Figure S2: PLS score plot of plasma samples of the three studied groups. Controls are clustered together on the negative part of the *y*-axis, NASH patients are grouped together on the positive side of the same axis and NAFL patients are scattered throughout the eclipse b. Permutation plot PLS model validation [Y Intercepts: R2 = (0.0, 0.229), Q2 = (0.0, −0.196)]. Figure S3: Optimal model validation test. Plot of permutation test results. Blue dots represent the performance of 200 models generated using the corresponding permuted response variables (NASH vs. control–NAFL), while the blue square represents the performance of the reference model generated using the intact response variable. Model performance is assessed using the Matthews Correlation Coefficient (MCC) metric. The *x*-axis corresponds to the correlation of each permuted response variable with the intact one, using Spearman’s correlation test. The performance of the test models is consistently significantly lower than that of the reference model, ruling out the possibility of random correlation of the predictor variable matrix with the response variable. Table S1: Summary of all identified lipids in the blood plasma of patients with NAFLD and controls. Information is provided regarding the lipid’s species, molecular formula, monoisotopic masses, the adducts and retention time for each lipid species. Table S2: Characteristics of the constructed unsupervised and supervised models. In models based on NAFLD groups pareto (PAR) scale was used.
