# Supplementary material for: Predicting Non-Alcoholic Steatohepatitis: A Lipidomics-Driven Machine Learning Approach
Source: Int J Mol Sci. 2024 May 29;25(11):5965. doi: 10.3390/ijms25115965 (PMC11172949; doi:10.3390/ijms25115965)
Supplement: Supplementary file 1 [file ijms-25-05965-s001.zip › ijms-2979648-supplementary.pdf]

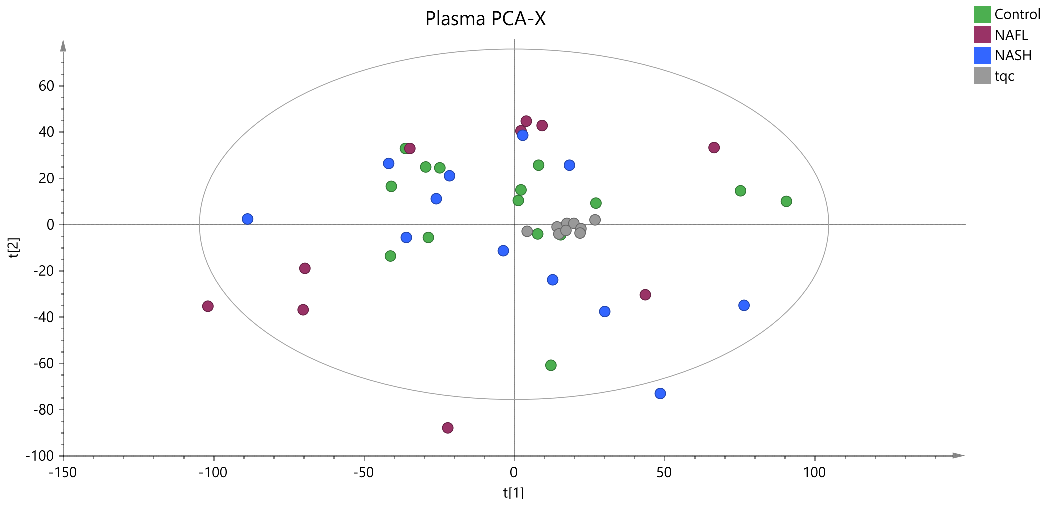

Supplementary Figure S1. PCA score plot for three studied groups and QC samples was constructed. Control group is illustrated in green, NAFL group in purple and NASH group in blue, whereas QC samples are depicted in grey and clustered together.

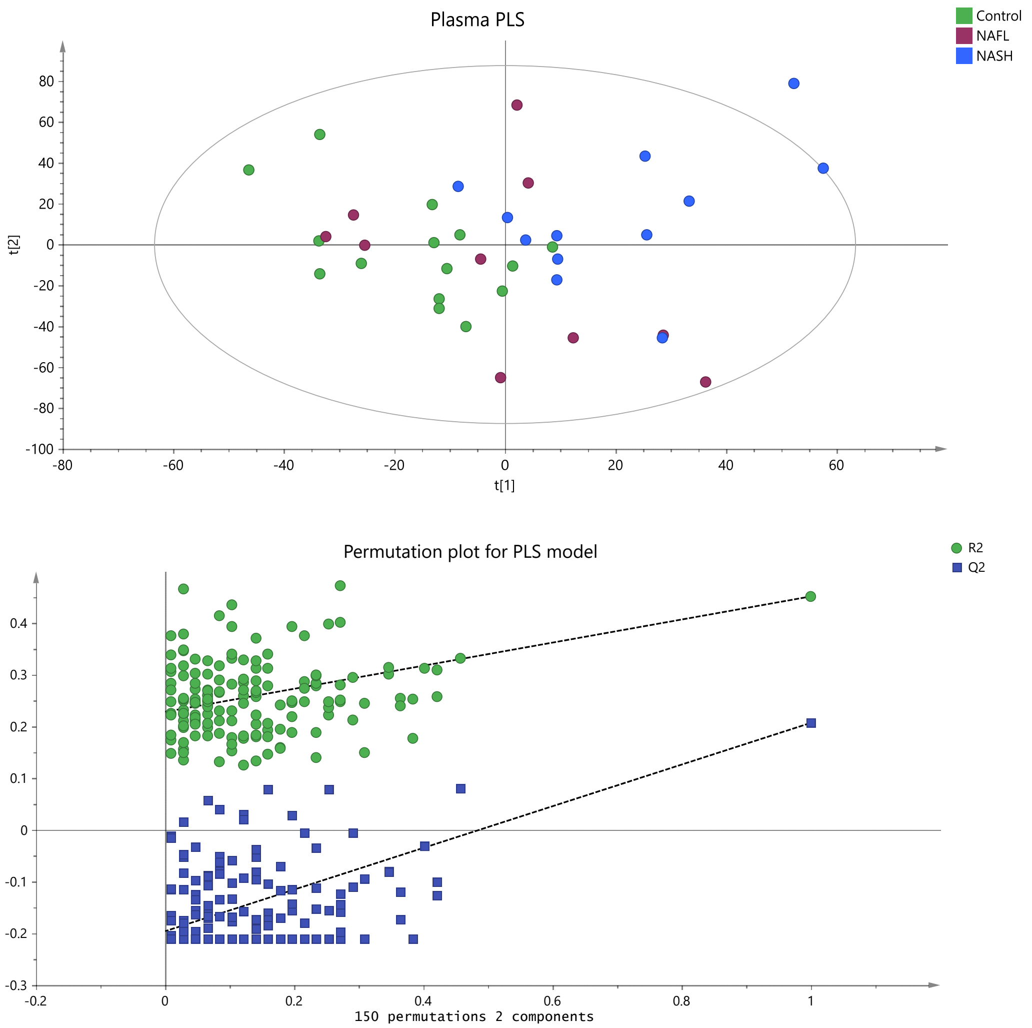

Supplementary Figure S2. PLS score plot of plasma samples of the three studied groups. Controls are clustered together on the negative part of the y-axis, NASH patients are grouped together on the positive side of the same axis and NAFL patients are scattered throughout the eclipse b. Permutation plot PLS model validation (Y Intercepts: R^2=(0.0, 0.229), Q^2=(0.0, -0.196))

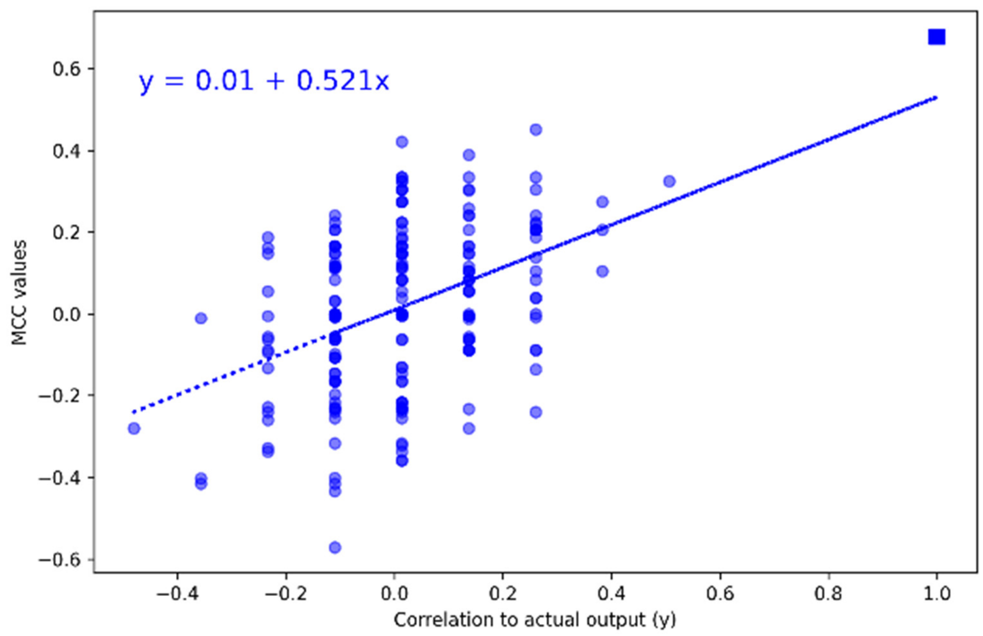

Supplementary Figure S3. Optimal model validation test. Plot of permutation test results. Blue dots represent the performance of 200 models generated using the corresponding permuted response variables (NASH vs control-NAFL), while the blue square represents the performance of the reference model generated using the intact response variable. Model performance is assessed using the Matthews Correlation Coefficient (MCC) metric. The x-axis corresponds to the correlation of each permuted response variable with the intact one, using Spearman's correlation test. The performance of the test models is consistently significantly lower than that of the reference model, ruling out the possibility of random correlation of the predictor variable matrix with the response variable.

Supplementary Table S1. Summary of all identified lipids in the blood plasma of patients with NAFLD and controls. Information is provided regarding the lipid's species, molecular formula, monoisotopic masses, the adducts and retention time for each lipid species.

Plasma											
Analysis Method	Lipids Subclasses	Lipids Species	Molecular Species	Molecular Formula	Monoisotopic Mass	$[M+H]^+$	$[M-H_2O+H]^+$	$[M+NH_4]^+$	$[M+HCOO]^-$	$[M-H]^-$	RT
**Fatty acids**											
GC-MS	FA	FA 12:0	FA 12:0	C12H24O2	200.1776						10.2
GC-MS	FA	FA 14:0	FA 14:0	C14H28O2	228.2089						12.4
GC-MS	FA	FA 15:0	FA 15:0	C15H30O2	242.2245						13.9
GC-MS	FA	FA 16:0	FA 16:0	C16H32O2	256.2402						15.7
GC-MS	FA	FA 17:0	FA 17:0	C17H34O2	270.2558						17.8
GC-MS	FA	FA 18:0	FA 18:0	C18H36O2	284.2715						20.0
GC-MS	FA	FA 20:0	FA 20:0	C20H40O2	312.3028						23.8
GC-MS	FA	FA 22:0	FA 22:0	C22H44O2	340.3341						27.8
GC-MS	FA	FA 24:0	FA 24:0	C24H48O2	368.3654						31.9
GC-MS	FA	FA 16:1	FA 16:1	C16H30O2	254.2245						17.1
GC-MS	FA	FA 18:1	FA 18:1	C18H34O2	282.2558						21.1
GC-MS	FA	FA 24:1	FA 24:1	C24H46O2	366.3497						33.0
GC-MS	FA	FA 20:1	FA 20:1	C20H38O2	310.2871						24.9
GC-MS	FA	FA 18:2	FA 18:2	C18H32O2	280.2402						22.8
GC-MS	FA	FA 20:5 ω3	FA 20:5 ω3	C20H30O2	302.2245						31.2
GC-MS	FA	FA 22:6 ω3	FA 22:6 ω3	C22H32O2	328.2402						36.1
GC-MS	FA	FA 18:3 ω3	FA 18:3 ω3	C18H30O2	278.2245						24.7
GC-MS	FA	FA 18:3 ω6	FA 18:3 ω6	C18H30O2	279.2245						23.9
GC-MS	FA	FA 20:3 ω6	FA 20:3 ω6	C20H34O2	306.2558						28.0
GC-MS	FA	FA 20:4 ω6	FA 20:4 ω6	C20H32O2	304.2402						29.1
**Acylcarnitines**											
HILIC-MS/MS	CAR	CAR 2:0	CAR 2:0	C9H17NO4	203.1157	$[M+H]^+$					10.0
HILIC-MS/MS	CAR	CAR 3:0	CAR 3:0	C10H19NO4	217.1314	$[M+H]^+$					8.5

HILIC-MS/MS	CAR	CAR 4:0	CAR 4:0	C11H21NO4	231.1470	[M+H]$^+$			7.4
HILIC-MS/MS	CAR	CAR 5:0	CAR 5:0	C12H23NO4	245.1627	[M+H]$^+$			6.6
HILIC-MS/MS	CAR	CAR 6:0	CAR 6:0	C13H25NO4	259.1783	[M+H]$^+$			6.0
HILIC-MS/MS	CAR	CAR 8:0	CAR 8:0	C15H29NO4	287.2096	[M+H]$^+$			5.2
HILIC-MS/MS	CAR	CAR 10:0	CAR 10:0	C17H33NO4	315.2409	[M+H]$^+$			4.7
HILIC-MS/MS	CAR	CAR 12:0	CAR 12:0	C19H37NO4	343.2722	[M+H]$^+$			4.3
HILIC-MS/MS	CAR	CAR 14:0	CAR 14:0	C21H41NO4	371.3035	[M+H]$^+$			4.0
HILIC-MS/MS	CAR	CAR 16:0	CAR 16:0	C23H45NO4	399.3348	[M+H]$^+$			3.8
HILIC-MS/MS	CAR	CAR 18:0	CAR 18:0	C25H49NO4	427.3661	[M+H]$^+$			3.5
HILIC-MS/MS	CAR	CAR 18:1	CAR 18:1	C25H47NO4	425.3505	[M+H]$^+$			3.6
HILIC-MS/MS	CAR	CAR 18:2	CAR 18:2	C25H45NO4	423.3348	[M+H]$^+$			3.8
**Ceramides**									
RPLC-MS/MS	Cer	Cer 34:1;O2	Cer 18:1;O2/16:0	C34H67NO3	537.5121	[M+H]$^+$	[M-H$_2$O+H]$^+$		1.9
RPLC-MS/MS	Cer	Cer 36:1;O2	Cer 18:1;O2/18:0	C36H71NO3	565.5434	[M+H]$^+$	[M-H$_2$O+H]$^+$		2.1
RPLC-MS/MS	Cer	Cer 42:1;O2	Cer 18:1;O2/24:0	C42H83NO3	649.6373	[M+H]$^+$	[M-H$_2$O+H]$^+$		2.5
RPLC-MS/MS	Cer	Cer 42:2;O2	Cer 18:1;O2/24:1	C42H81NO3	647.6216	[M+H]$^+$	[M-H$_2$O+H]$^+$		2.4
Lipidomics	Cer	Cer 40:0;O2	Cer 18:0;O2_22:0	C40H81NO3	623.6216	[M+H]$^+$	[M-H$_2$O+H]$^+$		21.1
Lipidomics	Cer	Cer 40:1;O2	Cer 18:1;O2_22:0	C40H79NO3	621.6060	[M+H]$^+$	[M-H$_2$O+H]$^+$		20.9
Lipidomics	Cer	Cer 41:1;O2	Cer 18:1;O2_23:0	C41H81NO3	635.6216	[M+H]$^+$	[M-H$_2$O+H]$^+$		21.2
Lipidomics	Hex2Cer	Hex2Cer 34:1;O2	Hex2Cer 18:1;O2/16:0	C46H87NO13	861.6177	[M+H]$^+$	[M-H$_2$O+H]$^+$		17.0
**Sphingomyelins**									
Lipidomics	SM	SM 30:1;O2	SM 30:1;O2	C35H71N2O6P	646.5050	[M+H]$^+$	[M-H$_2$O+H]$^+$	[M+HCOO]$^-$	14.2
Lipidomics	SM	SM 32:0;O2	SM 32:0;O2	C37H77N2O6P	676.5363	[M+H]$^+$	[M-H$_2$O+H]$^+$		16.2
Lipidomics	SM	SM 32:1;O2	SM 32:1;O2	C37H75N2O6P	674.5363	[M+H]$^+$	[M-H$_2$O+H]$^+$		15.7
Lipidomics	SM	SM 32:2;O2	SM 32:2;O2	C37H73N2O6P	672.5206	[M+H]$^+$	[M-H$_2$O+H]$^+$		14.5
Lipidomics	SM	SM 33:1;O2	SM 33:1;O2	C38H77N2O6P	688.5519	[M+H]$^+$	[M-H$_2$O+H]$^+$		16.3
Lipidomics	SM	SM 33:2;O2	SM 33:2;O2	C38H75N2O6P	686.5363	[M+H]$^+$	[M-H$_2$O+H]$^+$		15.3
Lipidomics	SM	SM 34:0;O2	SM 34:0;O2	C39H81N2O6P	704.5832	[M+H]$^+$	[M-H$_2$O+H]$^+$		17.4
Lipidomics	SM	SM 34:1;O2	SM 34:1;O2	C39H79N2O6P	702.5676	[M+H]$^+$	[M-H$_2$O+H]$^+$		16.9

Lipidomics	SM	SM 34:2;O2	SM 34:2;O2	C39H77N2O6P	700.5519	$[M+H]^+$	$[M-H_2O+H]^+$		15.9
Lipidomics	SM	SM 35:1;O2	SM 35:1;O2	C40H81N2O6P	716.5832	$[M+H]^+$	$[M-H_2O+H]^+$		17.5
Lipidomics	SM	SM 36:0;O2	SM 36:0;O2	C41H85N2O6P	732.6145	$[M+H]^+$	$[M-H_2O+H]^+$		18.4
Lipidomics	SM	SM 36:1;O2	SM 36:1;O2	C41H83N2O6P	730.5989	$[M+H]^+$	$[M-H_2O+H]^+$		18.1
Lipidomics	SM	SM 36:2;O2	SM 36:2;O2	C41H81N2O6P	728.5832	$[M+H]^+$	$[M-H_2O+H]^+$		17.2
Lipidomics	SM	SM 37:1;O2	SM 37:1;O2	C42H85N2O6P	744.6145	$[M+H]^+$	$[M-H_2O+H]^+$		18.6
Lipidomics	SM	SM 38:0;O2	SM 38:0;O2	C43H89N2O6P	760.6458	$[M+H]^+$	$[M-H_2O+H]^+$		19.4
Lipidomics	SM	SM 38:1;O2	SM 38:1;O2	C43H87N2O6P	758.6302	$[M+H]^+$	$[M-H_2O+H]^+$		19.0
Lipidomics	SM	SM 38:2;O2	SM 38:2;O2	C43H85N2O6P	756.6145	$[M+H]^+$	$[M-H_2O+H]^+$		18.2
Lipidomics	SM	SM 39:1;O2	SM 39:1;O2	C44H89N2O6P	772.6458	$[M+H]^+$	$[M-H_2O+H]^+$		19.5
Lipidomics	SM	SM 39:2;O2	SM 39:2;O2	C44H87N2O6P	770.6302	$[M+H]^+$	$[M-H_2O+H]^+$		18.7
Lipidomics	SM	SM 40:0;O2	SM 40:0;O2	C45H93N2O6P	788.6771	$[M+H]^+$	$[M-H_2O+H]^+$		20.2
Lipidomics	SM	SM 40:1;O2	SM 40:1;O2	C45H91N2O6P	786.6615	$[M+H]^+$	$[M-H_2O+H]^+$		19.9
Lipidomics	SM	SM 40:2;O2	SM 40:2;O2	C45H89N2O6P	784.6458	$[M+H]^+$	$[M-H_2O+H]^+$		19.2
Lipidomics	SM	SM 40:3;O2	SM 40:3;O2	C45H87N2O6P	782.6302	$[M+H]^+$	$[M-H_2O+H]^+$		18.3
Lipidomics	SM	SM 41:1;O2	SM 41:1;O2	C46H93N2O6P	800.6771	$[M+H]^+$	$[M-H_2O+H]^+$		20.3
Lipidomics	SM	SM 41:2;O2	SM 41:2;O2	C46H91N2O6P	798.6615	$[M+H]^+$	$[M-H_2O+H]^+$		19.4
Lipidomics	SM	SM 42:1;O2	SM 42:1;O2	C47H95N2O6P	814.6928	$[M+H]^+$	$[M-H_2O+H]^+$		20.6
Lipidomics	SM	SM 42:2;O2	SM 42:2;O2	C47H93N2O6P	812.6771	$[M+H]^+$	$[M-H_2O+H]^+$		19.8
Lipidomics	SM	SM 42:3;O2	SM 42:3;O2	C47H91N2O6P	810.6615	$[M+H]^+$	$[M-H_2O+H]^+$		19.1
Lipidomics	SM	SM 43:1;O2	SM 43:1;O2	C48H97N2O6P	828.7084	$[M+H]^+$	$[M-H_2O+H]^+$		20.8
Lipidomics	SM	SM 43:2;O2	SM 43:2;O2	C48H95N2O6P	826.6928	$[M+H]^+$	$[M-H_2O+H]^+$		20.1
**Phospholipids**									
**LPC**									
Lipidomics	LPC	LPC 14:0	LPC 14:0	C22H46NO7P	467.3012	$[M+H]^+$		$[M+HCOO]^-$	4.0
Lipidomics	LPC	LPC 15:0	LPC 15:0	C23H48NO7P	481.3168	$[M+H]^+$			5.2
Lipidomics	LPC	LPC 16:0	LPC 16:0	C24H50NO7P	495.3325	$[M+H]^+$		$[M+HCOO]^-$	6.7
Lipidomics	LPC	LPC 16:1	LPC 16:1	C24H48NO7P	493.3168	$[M+H]^+$		$[M+HCOO]^-$	4.6
Lipidomics	LPC	LPC 17:0	LPC 17:0	C25H52NO7P	509.3481	$[M+H]^+$		$[M+HCOO]^-$	8.1
Lipidomics	LPC	LPC 18:0	LPC 18:0	C26H54NO7P	523.3638	$[M+H]^+$		$[M+HCOO]^-$	9.4

Lipidomics	LPC	LPC 18:1	LPC 18:1	C26H52NO7P	521.3481	$[M+H]^+$	$[M+HCOO]^-$		7.3
Lipidomics	LPC	LPC 18:2	LPC 18:2	C26H50NO7P	519.3325	$[M+H]^+$	$[M+HCOO]^-$		5.5
Lipidomics	LPC	LPC 20:1	LPC 20:1	C28H56NO7P	549.3794	$[M+H]^+$	$[M+HCOO]^-$		9.8
Lipidomics	LPC	LPC 20:2	LPC 20:2	C28H54NO7P	547.3638	$[M+H]^+$	$[M+HCOO]^-$		8.0
Lipidomics	LPC	LPC 20:3	LPC 20:3	C28H52NO7P	545.3481	$[M+H]^+$	$[M+HCOO]^-$		6.4
Lipidomics	LPC	LPC 20:4	LPC 20:4	C28H50NO7P	543.3325	$[M+H]^+$	$[M+HCOO]^-$		5.4
Lipidomics	LPC	LPC 20:5	LPC 20:5	C28H48NO7P	541.3168	$[M+H]^+$	$[M+HCOO]^-$		4.0
Lipidomics	LPC	LPC 22:5	LPC 22:5	C30H52NO7P	569.3481	$[M+H]^+$	$[M+HCOO]^-$		5.9
Lipidomics	LPC	LPC 22:6	LPC 22:6	C30H50NO7P	567.3325	$[M+H]^+$	$[M+HCOO]^-$		5.2
Lipidomics	LPC	LPC P-16:0	LPC O-16:1	C24H50NO6P	479.3376	$[M+H]^+$	$[M+HCOO]^-$		7.6
Lipidomics	LPC	LPC O-18:1	LPC O-18:1	C26H54NO6P	507.3689	$[M+H]^+$			8.3
**LPE**									
Lipidomics	LPE	LPE 16:0	LPE 16:0	C21H44NO7P	453.2855	$[M+H]^+$		$[M-H]^-$	6.9
Lipidomics	LPE	LPE 18:0	LPE 18:0	C23H48NO7P	481.3168	$[M+H]^+$		$[M-H]^-$	9.7
Lipidomics	LPE	LPE 18:1	LPE 18:1	C23H46NO7P	479.3012	$[M+H]^+$		$[M-H]^-$	7.6
Lipidomics	LPE	LPE 18:2	LPE 18:2	C23H44NO7P	477.2855	$[M+H]^+$		$[M-H]^-$	5.6
Lipidomics	LPE	LPE 20:4	LPE 20:4	C25H44NO7P	501.2855	$[M+H]^+$		$[M-H]^-$	5.5
Lipidomics	LPE	LPE 22:6	LPE 22:6	C27H44NO7P	525.2855	$[M+H]^+$		$[M-H]^-$	5.3
**PC**									
Lipidomics	PC	PC 32:0	PC 16:0_16:0	C40H80NO8P	733.5622	$[M+H]^+$	$[M+HCOO]^-$		18.1
Lipidomics	PC	PC 32:1	PC 16:0_16:1	C40H78NO8P	731.5465	$[M+H]^+$	$[M+HCOO]^-$		17.2
Lipidomics	PC	PC 32:2	PC 14:0_18:2	C40H76NO8P	729.5309	$[M+H]^+$	$[M+HCOO]^-$		16.3
Lipidomics	PC	PC 33:1	PC 15:0_18:1	C41H80NO8P	745.5622	$[M+H]^+$	$[M+HCOO]^-$		17.7
Lipidomics	PC	PC 33:2	PC 15:0_18:2	C41H78NO8P	743.5465	$[M+H]^+$	$[M+HCOO]^-$		16.9
Lipidomics	PC	PC 34:0	PC 16:0_18:0	C42H84NO8P	761.5935	$[M+H]^+$	$[M+HCOO]^-$		19.0
Lipidomics	PC	PC 34:1	PC 16:0_18:1	C42H82NO8P	759.5778	$[M+H]^+$	$[M+HCOO]^-$		18.2
Lipidomics	PC	PC 34:2	PC 16:0_18:2	C42H80NO8P	757.5622	$[M+H]^+$	$[M+HCOO]^-$		17.4
Lipidomics	PC	PC 34:3	PC 16:1_18:2	C42H78NO8P	755.5465	$[M+H]^+$	$[M+HCOO]^-$		16.5
Lipidomics	PC	PC 34:3	PC 16:0_18:3	C42H78NO8P	755.5465	$[M+H]^+$	$[M+HCOO]^-$		16.7
Lipidomics	PC	PC 34:4	PC 14:0_20:4	C42H76NO8P	753.5309	$[M+H]^+$	$[M+HCOO]^-$		16.2

Lipidomics	PC	PC 35:1	PC 17:0_18:1	C43H84NO8P	773.5935	[M+H]$^+$	[M+HCOO]$^-$	18.6
Lipidomics	PC	PC 35:2	PC 17:1_18:1	C43H82NO8P	771.5778	[M+H]$^+$	[M+HCOO]$^-$	17.8
Lipidomics	PC	PC 35:2	PC 17:0_18:2	C43H82NO8P	771.5778	[M+H]$^+$	[M+HCOO]$^-$	17.9
Lipidomics	PC	PC 35:4	PC 15:0_20:4	C43H78NO8P	767.5465	[M+H]$^+$	[M+HCOO]$^-$	16.7
Lipidomics	PC	PC 36:1	PC 18:0_18:1	C44H86NO8P	787.6091	[M+H]$^+$	[M+HCOO]$^-$	19.1
Lipidomics	PC	PC 36:2	PC 18:1_18:1	C44H84NO8P	785.5935	[M+H]$^+$	[M+HCOO]$^-$	18.4
Lipidomics	PC	PC 36:2	PC 18:0_18:2	C44H84NO8P	785.5935	[M+H]$^+$	[M+HCOO]$^-$	17.5
Lipidomics	PC	PC 36:4	PC 16:0_20:4	C44H80NO8P	781.5622	[M+H]$^+$	[M+HCOO]$^-$	17.3
Lipidomics	PC	PC 36:5	PC 16:0_20:5	C44H78NO8P	779.5465	[M+H]$^+$	[M+HCOO]$^-$	16.6
Lipidomics	PC	PC 36:6	PC 14:0_22:6	C44H76NO8P	777.5309	[M+H]$^+$	[M+HCOO]$^-$	15.9
Lipidomics	PC	PC 37:4	PC 17:0_20:4	C45H82NO8P	795.5778	[M+H]$^+$	[M+HCOO]$^-$	17.8
Lipidomics	PC	PC 37:6	PC 15:0_22:6	C45H78NO8P	836.5447	[M+H]$^+$	[M+HCOO]$^-$	16.5
Lipidomics	PC	PC 38:3	PC 18:0_20:3	C46H86NO8P	811.6091	[M+H]$^+$	[M+HCOO]$^-$	18.7
Lipidomics	PC	PC 38:4	PC 18:1_20:3	C46H84NO8P	809.5935	[M+H]$^+$	[M+HCOO]$^-$	17.8
Lipidomics	PC	PC 38:4	PC 16:0_22:4	C46H84NO8P	809.5935	[M+H]$^+$	[M+HCOO]$^-$	18.0
Lipidomics	PC	PC 38:4	PC 18:0_20:4	C46H84NO8P	809.5935	[M+H]$^+$	[M+HCOO]$^-$	18.3
Lipidomics	PC	PC 38:5	PC 18:1_20:4	C46H82NO8P	807.5778	[M+H]$^+$	[M+HCOO]$^-$	17.4
Lipidomics	PC	PC 38:5	PC 18:0_20:5	C46H82NO8P	807.5778	[M+H]$^+$	[M+HCOO]$^-$	17.7
Lipidomics	PC	PC 38:6	PC 16:0_22:6	C46H80NO8P	805.5622	[M+H]$^+$	[M+HCOO]$^-$	17.0
Lipidomics	PC	PC 40:4	PC 18:0_22:4	C48H88NO8P	837.6248	[M+H]$^+$	[M+HCOO]$^-$	18.9
Lipidomics	PC	PC 40:5	PC 18:0_22:5	C48H86NO8P	835.6091	[M+H]$^+$	[M+HCOO]$^-$	18.3
Lipidomics	PC	PC 40:6	PC 18:0_22:6	C48H84NO8P	833.5935	[M+H]$^+$	[M+HCOO]$^-$	18.1
Lipidomics	PC	PC P-34:2	PC P-16:0_18:2	C42H80NO7P	741.5672	[M+H]$^+$	[M+HCOO]$^-$	17.9
Lipidomics	PC	PC O-36:4	PC O-16:0_20:4	C44H82NO7P	767.5829	[M+H]$^+$	[M+HCOO]$^-$	17.9
Lipidomics	PC	PC P-36:4	PC P-16:0_20:4	C44H80NO7P	765.5672	[M+H]$^+$	[M+HCOO]$^-$	17.7
Lipidomics	PC	PC O-38:5	PC O-18:1_20:4	C46H84NO7P	793.5985	[M+H]$^+$	[M+HCOO]$^-$	18.0
Lipidomics	PC	PC 35:3		C43H80NO8P	769.5622	[M+H]$^+$		17.0
Lipidomics	PC	PC 35:5		C43H76NO8P	765.5308	[M+H]$^+$		16.0
Lipidomics	PC	PC 37:3		C45H84NO8P	797.5935	[M+H]$^+$		18.2
Lipidomics	PC	PC 38:2		C46H88NO8P	813.6248	[M+H]$^+$		19.2

Lipidomics	PC	PC 38:8		C46H76NO8P	801.5308	[M+H]$^+$		16.6
Lipidomics	PC	PC 39:4		C47H86NO8P	823.6091	[M+H]$^+$		18.8
Lipidomics	PC	PC 39:6		C47H82NO8P	819.5778	[M+H]$^+$		17.4
Lipidomics	PC	PC 40:9		C48H78NO8P	827.5465	[M+H]$^+$		17.0
Lipidomics	PC	PC 42:9		C50H82NO8P	855.5778	[M+H]$^+$		18.1
				**PE**				
Lipidomics	PE	PE 34:1	PE 16:0_18:1	C39H76NO8P	717.5309	[M+H]$^+$	[M-H]$^-$	18.5
Lipidomics	PE	PE 34:2	PE 16:0_18:2	C39H74NO8P	715.5152	[M+H]$^+$	[M-H]$^-$	17.7
Lipidomics	PE	PE 36:1	PE 18:0_18:1	C41H80NO8P	745.5622	[M+H]$^+$	[M-H]$^-$	19.3
Lipidomics	PE	PE 36:2	PE 18:1_18:1	C41H78NO8P	743.5465	[M+H]$^+$	[M-H]$^-$	18.7
Lipidomics	PE	PE 36:2	PE 18:0_18:2	C41H78NO8P	743.5465	[M+H]$^+$	[M-H]$^-$	18.7
Lipidomics	PE	PE 36:4	PE 18:2_18:2	C41H74NO8P	739.5152	[M+H]$^+$	[M-H]$^-$	17.6
Lipidomics	PE	PE 36:4	PE 16:0_20:4	C41H74NO8P	739.5152	[M+H]$^+$	[M-H]$^-$	17.6
Lipidomics	PE	PE 38:3	PE 18:0_20:3	C43H80NO8P	769.5622	[M+H]$^+$	[M-H]$^-$	18.9
Lipidomics	PE	PE 38:4	PE 18:0_20:4	C43H78NO8P	767.5465	[M+H]$^+$	[M-H]$^-$	18.6
Lipidomics	PE	PE 38:5	PE 16:0_22:5	C43H76NO8P	765.5309	[M+H]$^+$	[M-H]$^-$	17.8
Lipidomics	PE	PE 38:5	PE 18:1_20:4	C43H76NO8P	765.5309	[M+H]$^+$	[M-H]$^-$	17.8
Lipidomics	PE	PE 38:6	PE 16:0_22:6	C43H74NO8P	763.5152	[M+H]$^+$	[M-H]$^-$	17.3
Lipidomics	PE	PE 40:6	PE 18:0_22:6	C45H78NO8P	791.5465	[M+H]$^+$	[M-H]$^-$	18.3
Lipidomics	PE	PE P-36:2	PE P-18:0_18:2	C41H78NO7P	727.5516	[M+H]$^+$	[M-H]$^-$	19.2
Lipidomics	PE	PE P-36:4	PE P-16:0_20:4	C41H74NO7P	723.5203	[M+H]$^+$	[M-H]$^-$	18.1
Lipidomics	PE	PE P-38:4	PE P-18:0_20:4	C43H78NO7P	751.5516	[M+H]$^+$	[M-H]$^-$	19.0
Lipidomics	PE	PE P-38:6	PE P-16:0_22:6	C43H74NO7P	747.5203	[M+H]$^+$	[M-H]$^-$	17.8
Lipidomics	PE	PE P-40:6	PE P-18:0_22:6	C45H78NO7P	775.5516	[M+H]$^+$		18.8
Lipidomics	PE	PE P-40:7	PE P-18:1_22:6	C45H76NO7P	773.5359	[M+H]$^+$	[M-H]$^-$	17.9
Lipidomics	PE	PE O-34:2		C39H76NO7P	701.5359	[M+H]$^+$	[M-H]$^-$	18.9
Lipidomics	PE	PE P-34:2		C39H74NO7P	699.5202	[M+H]$^+$	[M-H]$^-$	18.2
Lipidomics	PE	PE O-36:2		C41H80NO7P	729.5672	[M+H]$^+$	[M-H]$^-$	19.8
Lipidomics	PE	PE O-38:4		C43H80NO7P	753.5672	[M+H]$^+$	[M-H]$^-$	19.4
Lipidomics	PE	PE O-40:5		C45H82NO7P	779.5828	[M+H]$^+$	[M-H]$^-$	19.8

**PI**									
Lipidomics	PI	PI 34:2	PI 16:1_18:1	C43H79O13P	834.5258	$[M+H]^+$		$[M-H]^-$	16.1
Lipidomics	PI	PI 36:2	PI 18:0_18:2	C45H83O13P	862.5571	$[M+H]^+$		$[M-H]^-$	17.2
Lipidomics	PI	PI 36:4	PI 16:0_20:4	C45H79O13P	858.5258	$[M+H]^+$		$[M-H]^-$	16.0
Lipidomics	PI	PI 38:4	PI 18:0_20:4	C47H83O13P	886.5571	$[M+H]^+$		$[M-H]^-$	17.1
**Neutral Lipids**									
**CE**									
Lipidomics	CE	CE 16:0	CE 16:0	C43H76O2	624.5845		$[M+NH_4]^+$		24.2
Lipidomics	CE	CE 16:1	CE 16:1	C43H74O2	622.5689		$[M+NH_4]^+$		23.7
Lipidomics	CE	CE 17:1	CE 17:1	C44H76O2	636.5845		$[M+NH_4]^+$		23.9
Lipidomics	CE	CE 18:0	CE 18:0	C45H80O2	652.6158		$[M+NH_4]^+$		24.9
Lipidomics	CE	CE 18:1	CE 18:1	C45H78O2	650.6002		$[M+NH_4]^+$		24.2
Lipidomics	CE	CE 18:2	CE 18:2	C45H76O2	648.5845		$[M+NH_4]^+$		23.7
Lipidomics	CE	CE 18:3	CE 18:3	C45H74O2	646.5689		$[M+NH_4]^+$		23.3
Lipidomics	CE	CE 20:3	CE 20:3	C47H78O2	674.6002		$[M+NH_4]^+$		23.8
Lipidomics	CE	CE 20:4	CE 20:4	C47H76O2	672.5845		$[M+NH_4]^+$		23.5
Lipidomics	CE	CE 20:5	CE 20:5	C47H74O2	670.5689		$[M+NH_4]^+$		23.1
Lipidomics	CE	CE 22:5	CE 22:5	C49H78O2	698.6002		$[M+NH_4]^+$		23.7
Lipidomics	CE	CE 22:6	CE 22:6	C49H76O2	696.5845		$[M+NH_4]^+$		23.3
**DG**									
Lipidomics	DG	DG 32:0	DG 16:0_16:0	C35H68O5	568.5067		$[M+NH_4]^+$		19.9
Lipidomics	DG	DG 34:1	DG 16:1_18:0	C37H70O5	594.5223		$[M+NH_4]^+$		20.0
Lipidomics	DG	DG 34:2	DG 16:0_18:2	C37H68O5	592.5067		$[M+NH_4]^+$		19.3
Lipidomics	DG	DG 36:0	DG 18:0_18:0	C39H76O5	624.5693		$[M+NH_4]^+$		21.3
Lipidomics	DG	DG 36:1	DG 18:0_18:1	C39H74O5	622.5536		$[M+NH_4]^+$		20.7
Lipidomics	DG	DG 36:2	DG 18:1_18:1	C39H72O5	620.5380		$[M+NH_4]^+$		20.0
Lipidomics	DG	DG 36:3	DG 18:1_18:2	C39H70O5	618.5223		$[M+NH_4]^+$		19.4
Lipidomics	DG	DG 36:4	DG 18:2_18:2	C39H68O5	616.5067		$[M+NH_4]^+$		18.7
Lipidomics	DG	DG 38:5	DG 18:1_20:4	C41H70O5	642.5223		$[M+NH_4]^+$		19.2
**TG**									

Lipidomics	TG	TG 40:0	TG 10:0_14:0_16:0	C43H82O6	694.6111	$[M+NH_4]^+$	22.1
Lipidomics	TG	TG 40:0	TG 12:0_14:0_14:0	C43H82O6	694.6111	$[M+NH_4]^+$	22.1
Lipidomics	TG	TG 41:0	TG 12:0_13:0_16:0	C44H84O6	708.6268	$[M+NH_4]^+$	22.3
Lipidomics	TG	TG 41:0	TG 12:0_14:0_15:0	C44H84O6	708.6268	$[M+NH_4]^+$	22.3
Lipidomics	TG	TG 41:0	TG 13:0_13:0_15:0	C44H84O6	708.6268	$[M+NH_4]^+$	22.3
Lipidomics	TG	TG 41:0	TG 13:0_14:0_14:0	C44H84O6	708.6268	$[M+NH_4]^+$	22.3
Lipidomics	TG	TG 42:0	TG 10:0_14:0_18:0	C45H86O6	722.6424	$[M+NH_4]^+$	22.6
Lipidomics	TG	TG 42:0	TG 10:0_16:0_16:0	C45H86O6	722.6424	$[M+NH_4]^+$	22.6
Lipidomics	TG	TG 42:0	TG 12:0_12:0_18:0	C45H86O6	722.6424	$[M+NH_4]^+$	22.6
Lipidomics	TG	TG 42:0	TG 12:0_14:0_16:0	C45H86O6	722.6424	$[M+NH_4]^+$	22.6
Lipidomics	TG	TG 42:0	TG 14:0_14:0_14:0	C45H86O6	722.6424	$[M+NH_4]^+$	22.6
Lipidomics	TG	TG 42:1	TG 10:0_14:0_18:1	C45H84O6	720.6268	$[M+NH_4]^+$	22.1
Lipidomics	TG	TG 42:1	TG 10:0_16:0_16:1	C45H84O6	720.6268	$[M+NH_4]^+$	22.1
Lipidomics	TG	TG 42:1	TG 12:0_12:0_18:1	C45H84O6	720.6268	$[M+NH_4]^+$	22.1
Lipidomics	TG	TG 42:1	TG 12:0_14:0_16:1	C45H84O6	720.6268	$[M+NH_4]^+$	22.1
Lipidomics	TG	TG 43:0	TG 12:0_15:0_16:0	C46H88O6	736.6581	$[M+NH_4]^+$	22.8
Lipidomics	TG	TG 43:0	TG 13:0_14:0_16:0	C46H88O6	736.6581	$[M+NH_4]^+$	22.8
Lipidomics	TG	TG 43:0	TG 13:0_15:0_15:0	C46H88O6	736.6581	$[M+NH_4]^+$	22.8
Lipidomics	TG	TG 43:0	TG 14:0_14:0_15:0	C46H88O6	736.6581	$[M+NH_4]^+$	22.8
Lipidomics	TG	TG 43:1	TG 13:0_14:0_16:1	C46H86O6	734.6424	$[M+NH_4]^+$	22.4
Lipidomics	TG	TG 43:2	TG 13:0_14:1_16:1	C46H84O6	732.6267	$[M+NH_4]^+$	22.0
Lipidomics	TG	TG 44:0	TG 12:0_14:0_18:0	C47H90O6	750.6737	$[M+NH_4]^+$	23.0
Lipidomics	TG	TG 44:0	TG 12:0_16:0_16:0	C47H90O6	750.6737	$[M+NH_4]^+$	23.0
Lipidomics	TG	TG 44:0	TG 14:0_14:0_16:0	C47H90O6	750.6737	$[M+NH_4]^+$	23.0
Lipidomics	TG	TG 44:0	TG 14:0_15:0_15:0	C47H90O6	750.6737	$[M+NH_4]^+$	23.0
Lipidomics	TG	TG 44:1	TG 10:0_16:0_18:1	C47H88O6	748.6581	$[M+NH_4]^+$	22.6
Lipidomics	TG	TG 44:1	TG 12:0_14:0_18:1	C47H88O6	748.6581	$[M+NH_4]^+$	22.6
Lipidomics	TG	TG 44:2	TG 10:0_16:1_18:1	C47H86O6	746.6424	$[M+NH_4]^+$	22.2
Lipidomics	TG	TG 44:2	TG 12:0_14:0_18:2	C47H86O6	746.6424	$[M+NH_4]^+$	22.2
Lipidomics	TG	TG 44:2	TG 12:0_14:1_18:1	C47H86O6	746.6424	$[M+NH_4]^+$	22.2

Lipidomics	TG	TG 44:2	TG 12:0_16:1_16:1	C47H86O6	746.6424	$[M+NH_4]^+$	22.2
Lipidomics	TG	TG 44:2	TG 14:0_14:1_16:1	C47H86O6	746.6424	$[M+NH_4]^+$	22.2
Lipidomics	TG	TG 44:2	TG 14:1_14:1_16:0	C47H86O6	746.6424	$[M+NH_4]^+$	22.2
Lipidomics	TG	TG 45:0	TG 12:0_16:0_17:0	C48H92O6	764.6894	$[M+NH_4]^+$	23.2
Lipidomics	TG	TG 45:0	TG 13:0_15:0_17:0	C48H92O6	764.6894	$[M+NH_4]^+$	23.2
Lipidomics	TG	TG 45:0	TG 13:0_16:0_16:0	C48H92O6	764.6894	$[M+NH_4]^+$	23.2
Lipidomics	TG	TG 45:0	TG 14:0_14:0_17:0	C48H92O6	764.6894	$[M+NH_4]^+$	23.2
Lipidomics	TG	TG 45:0	TG 14:0_15:0_16:0	C48H92O6	764.6894	$[M+NH_4]^+$	23.2
Lipidomics	TG	TG 45:0	TG 15:0_15:0_15:0	C48H92O6	764.6894	$[M+NH_4]^+$	23.2
Lipidomics	TG	TG 45:1	TG 12:0_15:0_18:1	C48H90O6	762.6737	$[M+NH_4]^+$	22.8
Lipidomics	TG	TG 45:1	TG 12:0_16:0_17:1	C48H90O6	762.6737	$[M+NH_4]^+$	22.8
Lipidomics	TG	TG 45:1	TG 13:0_14:0_18:1	C48H90O6	762.6737	$[M+NH_4]^+$	22.8
Lipidomics	TG	TG 45:1	TG 13:0_16:0_16:1	C48H90O6	762.6737	$[M+NH_4]^+$	22.8
Lipidomics	TG	TG 45:1	TG 14:0_14:0_17:1	C48H90O6	762.6737	$[M+NH_4]^+$	22.8
Lipidomics	TG	TG 45:1	TG 14:0_15:0_16:1	C48H90O6	762.6737	$[M+NH_4]^+$	22.8
Lipidomics	TG	TG 45:1	TG 14:0_15:1_16:0	C48H90O6	762.6737	$[M+NH_4]^+$	22.8
Lipidomics	TG	TG 45:1	TG 15:0_15:0_15:1	C48H90O6	762.6737	$[M+NH_4]^+$	22.8
Lipidomics	TG	TG 45:2	TG 14:0_15:1_16:1	C48H88O6	760.6581	$[M+NH_4]^+$	22.5
Lipidomics	TG	TG 45:2	TG 14:1_15:0_16:1	C48H88O6	760.6581	$[M+NH_4]^+$	22.5
Lipidomics	TG	TG 45:2	TG 14:1_15:1_16:0	C48H88O6	760.6581	$[M+NH_4]^+$	22.5
Lipidomics	TG	TG 45:2	TG 15:0_15:1_15:1	C48H88O6	760.6581	$[M+NH_4]^+$	22.5
Lipidomics	TG	TG 45:3	TG 14:1_15:1_16:1	C48H86O6	758.6424	$[M+NH_4]^+$	22.1
Lipidomics	TG	TG 46:0	TG 14:0_14:0_18:0	C49H94O6	778.7050	$[M+NH_4]^+$	23.4
Lipidomics	TG	TG 46:0	TG 14:0_16:0_16:0	C49H94O6	778.7050	$[M+NH_4]^+$	23.4
Lipidomics	TG	TG 46:1	TG 12:0_16:0_18:1	C49H92O6	776.6894	$[M+NH_4]^+$	23.0
Lipidomics	TG	TG 46:1	TG 14:0_14:0_18:1	C49H92O6	776.6894	$[M+NH_4]^+$	23.0
Lipidomics	TG	TG 46:1	TG 14:0_16:0_16:1	C49H92O6	776.6894	$[M+NH_4]^+$	23.0
Lipidomics	TG	TG 46:2	TG 10:0_18:1_18:1	C49H90O6	774.6737	$[M+NH_4]^+$	22.6
Lipidomics	TG	TG 46:2	TG 12:0_16:0_18:2	C49H90O6	774.6737	$[M+NH_4]^+$	22.6
Lipidomics	TG	TG 46:2	TG 12:0_16:1_18:1	C49H90O6	774.6737	$[M+NH_4]^+$	22.6

Lipidomics	TG	TG 46:2	TG 14:0_14:0_18:2	C49H90O6	774.6737	$[M+NH_4]^+$	22.6
Lipidomics	TG	TG 46:2	TG 14:0_14:1_18:1	C49H90O6	774.6737	$[M+NH_4]^+$	22.6
Lipidomics	TG	TG 46:2	TG 14:0_16:1_16:1	C49H90O6	774.6737	$[M+NH_4]^+$	22.6
Lipidomics	TG	TG 46:2	TG 14:1_16:0_16:1	C49H90O6	774.6737	$[M+NH_4]^+$	22.6
Lipidomics	TG	TG 47:1	TG 14:0_15:0_18:1	C50H94O6	790.7050	$[M+NH_4]^+$	23.2
Lipidomics	TG	TG 47:1	TG 14:0_16:0_17:1	C50H94O6	790.7050	$[M+NH_4]^+$	23.2
Lipidomics	TG	TG 47:1	TG 14:0_16:1_17:0	C50H94O6	790.7050	$[M+NH_4]^+$	23.2
Lipidomics	TG	TG 47:1	TG 14:1_16:0_17:0	C50H94O6	790.7050	$[M+NH_4]^+$	23.2
Lipidomics	TG	TG 47:1	TG 15:0_15:0_17:1	C50H94O6	790.7050	$[M+NH_4]^+$	23.2
Lipidomics	TG	TG 47:1	TG 15:0_16:0_16:1	C50H94O6	790.7050	$[M+NH_4]^+$	23.2
Lipidomics	TG	TG 47:1	TG 15:1_16:0_16:0	C50H94O6	790.7050	$[M+NH_4]^+$	23.2
Lipidomics	TG	TG 47:2	TG 14:0_15:0_18:2	C50H92O6	788.6894	$[M+NH_4]^+$	22.9
Lipidomics	TG	TG 47:2	TG 14:0_15:1_18:1	C50H92O6	788.6894	$[M+NH_4]^+$	22.9
Lipidomics	TG	TG 47:2	TG 14:0_16:1_17:1	C50H92O6	788.6894	$[M+NH_4]^+$	22.9
Lipidomics	TG	TG 47:2	TG 14:1_15:0_18:1	C50H92O6	788.6894	$[M+NH_4]^+$	22.9
Lipidomics	TG	TG 47:3	TG 14:1_16:0_17:1	C50H90O6	786.6737	$[M+NH_4]^+$	22.6
Lipidomics	TG	TG 47:2	TG 15:0_15:1_17:1	C50H92O6	788.6894	$[M+NH_4]^+$	22.9
Lipidomics	TG	TG 47:2	TG 15:0_16:1_16:1	C50H92O6	788.6894	$[M+NH_4]^+$	22.9
Lipidomics	TG	TG 47:2	TG 15:1_16:0_16:1	C50H92O6	788.6894	$[M+NH_4]^+$	22.9
Lipidomics	TG	TG 47:3	TG 15:0_16:1_18:2	C50H90O6	786.6737	$[M+NH_4]^+$	22.6
Lipidomics	TG	TG 48:0	TG 14:0_16:0_18:2	C51H98O6	806.7363	$[M+NH_4]^+$	23.8
Lipidomics	TG	TG 48:1	TG 14:0_16:0_18:1	C51H96O6	804.7207	$[M+NH_4]^+$	23.4
Lipidomics	TG	TG 48:1	TG 16:0_16:0_16:1	C51H96O6	804.7207	$[M+NH_4]^+$	23.4
Lipidomics	TG	TG 48:2	TG 12:0_18:1_18:1	C51H94O6	802.7050	$[M+NH_4]^+$	23.1
Lipidomics	TG	TG 48:2	TG 14:0_16:0_18:2	C51H94O6	802.7050	$[M+NH_4]^+$	23.1
Lipidomics	TG	TG 48:2	TG 14:0_16:1_18:1	C51H94O6	802.7050	$[M+NH_4]^+$	23.1
Lipidomics	TG	TG 48:2	TG 16:0_16:1_16:1	C51H94O6	802.7050	$[M+NH_4]^+$	23.1
Lipidomics	TG	TG 48:3	TG 12:0_18:1_18:2	C51H92O6	800.6894	$[M+NH_4]^+$	22.7
Lipidomics	TG	TG 48:3	TG 12:1_18:1_18:1	C51H92O6	800.6894	$[M+NH_4]^+$	22.7
Lipidomics	TG	TG 48:3	TG 14:0_16:1_18:2	C51H92O6	800.6894	$[M+NH_4]^+$	22.7

Lipidomics	TG	TG 48:3	TG 14:1_16:0_18:2	C51H92O6	800.6894	$[M+NH_4]^+$	22.7
Lipidomics	TG	TG 48:3	TG 14:1_16:1_18:1	C51H92O6	800.6894	$[M+NH_4]^+$	22.7
Lipidomics	TG	TG 48:3	TG 16:1_16:1_16:1	C51H92O6	800.6894	$[M+NH_4]^+$	22.7
Lipidomics	TG	TG 48:4	TG 12:0_18:2_18:2	C51H90O6	798.6737	$[M+NH_4]^+$	22.3
Lipidomics	TG	TG 49:0	TG 15:0_16:0_18:0	C52H100O6	820.7520	$[M+NH_4]^+$	24.0
Lipidomics	TG	TG 49:1	TG 14:0_17:0_18:1	C52H98O6	818.7363	$[M+NH_4]^+$	23.6
Lipidomics	TG	TG 49:1	TG 15:0_16:0_18:1	C52H98O6	818.7363	$[M+NH_4]^+$	23.6
Lipidomics	TG	TG 49:1	TG 15:0_17:0_17:1	C52H98O6	818.7363	$[M+NH_4]^+$	23.6
Lipidomics	TG	TG 49:2	TG 15:0_16:0_18:2	C52H96O6	816.7207	$[M+NH_4]^+$	23.3
Lipidomics	TG	TG 49:2	TG 15:0_16:1_18:1	C52H96O6	816.7207	$[M+NH_4]^+$	23.3
Lipidomics	TG	TG 49:2	TG 15:0_17:1_17:1	C52H96O6	816.7207	$[M+NH_4]^+$	23.3
Lipidomics	TG	TG 49:2	TG 16:0_16:1_17:1	C52H96O6	816.7207	$[M+NH_4]^+$	23.3
Lipidomics	TG	TG 49:2	TG 16:1_16:1_17:0	C52H96O6	816.7207	$[M+NH_4]^+$	23.3
Lipidomics	TG	TG 49:3	TG 14:0_17:1_18:2	C52H94O6	814.7050	$[M+NH_4]^+$	22.9
Lipidomics	TG	TG 49:3	TG 15:0_16:1_18:2	C52H94O6	814.7050	$[M+NH_4]^+$	22.9
Lipidomics	TG	TG 49:3	TG 15:1_16:0_18:2	C52H94O6	814.7050	$[M+NH_4]^+$	22.9
Lipidomics	TG	TG 49:3	TG 15:1_16:1_18:1	C52H94O6	814.7050	$[M+NH_4]^+$	22.9
Lipidomics	TG	TG 49:3	TG 15:1_17:1_17:1	C52H94O6	814.7050	$[M+NH_4]^+$	22.9
Lipidomics	TG	TG 49:3	TG 16:1_16:1_17:1	C52H94O6	814.7050	$[M+NH_4]^+$	22.9
Lipidomics	TG	TG 50:0	TG 16:0_16:0_18:0	C53H102O6	834.7676	$[M+NH_4]^+$	24.3
Lipidomics	TG	TG 50:1	TG 16:0_16:0_18:1	C53H100O6	832.7520	$[M+NH_4]^+$	23.8
Lipidomics	TG	TG 50:2	TG 14:0_18:1_18:1	C53H98O6	830.7363	$[M+NH_4]^+$	23.4
Lipidomics	TG	TG 50:2	TG 16:0_16:1_18:1	C53H98O6	830.7363	$[M+NH_4]^+$	23.4
Lipidomics	TG	TG 50:3	TG 14:0_18:1_18:2	C53H96O6	828.7207	$[M+NH_4]^+$	23.1
Lipidomics	TG	TG 50:3	TG 16:0_16:1_18:2	C53H96O6	828.7207	$[M+NH_4]^+$	23.1
Lipidomics	TG	TG 50:3	TG 16:1_16:1_18:1	C53H96O6	828.7207	$[M+NH_4]^+$	23.1
Lipidomics	TG	TG 50:4	TG 16:1_16:1_18:2	C53H94O6	826.7050	$[M+NH_4]^+$	22.8
Lipidomics	TG	TG 50:5	TG 14:0_18:2_18:3	C53H92O6	824.6894	$[M+NH_4]^+$	22.4
Lipidomics	TG	TG 50:5	TG 16:0_16:2_18:3	C53H92O6	824.6894	$[M+NH_4]^+$	22.4
Lipidomics	TG	TG 50:5	TG 16:1_16:2_18:2	C53H92O6	824.6894	$[M+NH_4]^+$	22.4

Lipidomics	TG	TG 51:0	TG 16:0_17:0_18:0	C54H104O6	848.7832	$[M+NH_4]^+$	24.5
Lipidomics	TG	TG 51:1	TG 16:0_17:0_18:1	C54H102O6	846.7676	$[M+NH_4]^+$	24.1
Lipidomics	TG	TG 51:2	TG 15:0_18:1_18:1	C54H102O6	844.7520	$[M+NH_4]^+$	23.6
Lipidomics	TG	TG 51:2	TG 16:0_17:1_18:1	C54H102O6	844.7520	$[M+NH_4]^+$	23.6
Lipidomics	TG	TG 51:3	TG 15:0_18:1_18:2	C54H98O6	842.7363	$[M+NH_4]^+$	23.3
Lipidomics	TG	TG 51:3	TG 16:0_17:1_18:2	C54H98O6	842.7363	$[M+NH_4]^+$	23.3
Lipidomics	TG	TG 51:3	TG 16:1_17:0_18:2	C54H98O6	842.7363	$[M+NH_4]^+$	23.3
Lipidomics	TG	TG 51:3	TG 17:1_17:1_17:1	C54H98O6	842.7363	$[M+NH_4]^+$	23.3
Lipidomics	TG	TG 51:4	TG 15:0_18:2_18:2	C54H96O6	840.7207	$[M+NH_4]^+$	23.0
Lipidomics	TG	TG 51:4	TG 16:1_17:1_18:2	C54H96O6	840.7207	$[M+NH_4]^+$	23.0
Lipidomics	TG	TG 52:1	TG 16:0_18:0_18:1	C55H104O6	860.7833	$[M+NH_4]^+$	24.3
Lipidomics	TG	TG 52:2	TG 16:0_18:0_18:2	C55H102O6	858.7676	$[M+NH_4]^+$	23.9
Lipidomics	TG	TG 52:2	TG 16:0_18:1_18:1	C55H102O6	858.7676	$[M+NH_4]^+$	23.9
Lipidomics	TG	TG 52:3	TG 16:0_18:1_18:2	C55H100O6	856.7520	$[M+NH_4]^+$	23.5
Lipidomics	TG	TG 52:4	TG 16:0_18:2_18:2	C55H98O6	854.7363	$[M+NH_4]^+$	23.2
Lipidomics	TG	TG 52:5	TG 16:0_18:2_18:3	C55H96O6	852.7207	$[M+NH_4]^+$	22.9
Lipidomics	TG	TG 52:5	TG 16:1_18:1_18:3	C55H96O6	852.7207	$[M+NH_4]^+$	22.9
Lipidomics	TG	TG 52:5	TG 16:1_18:2_18:2	C55H96O6	852.7207	$[M+NH_4]^+$	22.9
Lipidomics	TG	TG 52:6	TG 16:0_18:3_18:3	C55H94O6	850.7050	$[M+NH_4]^+$	22.6
Lipidomics	TG	TG 52:6	TG 16:1_18:2_18:3	C55H94O6	850.7050	$[M+NH_4]^+$	22.6
Lipidomics	TG	TG 53:2	TG 17:0_18:1_18:1	C56H104O6	872.7833	$[M+NH_4]^+$	24.1
Lipidomics	TG	TG 53:3	TG 17:1_18:1_18:1	C56H102O6	870.7676	$[M+NH_4]^+$	23.7
Lipidomics	TG	TG 53:4	TG 17:1_18:1_18:2	C56H100O6	868.7520	$[M+NH_4]^+$	23.3
Lipidomics	TG	TG 53:5	TG 17:1_18:2_18:2	C56H98O6	866.7363	$[M+NH_4]^+$	23.0
Lipidomics	TG	TG 54:0	TG 16:0_16:0_22:0	C57H110O6	890.8302	$[M+NH_4]^+$	25.4
Lipidomics	TG	TG 54:1	TG 16:0_18:1_20:0	C57H108O6	888.8146	$[M+NH_4]^+$	24.8
Lipidomics	TG	TG 54:1	TG 18:0_18:0_18:1	C57H108O6	888.8146	$[M+NH_4]^+$	24.8
Lipidomics	TG	TG 54:2	TG 16:0_18:1_20:1	C57H106O6	886.7989	$[M+NH_4]^+$	24.3
Lipidomics	TG	TG 54:2	TG 18:0_18:1_18:1	C57H106O6	886.7989	$[M+NH_4]^+$	24.3
Lipidomics	TG	TG 54:3	TG 16:0_18:0_20:3	C57H104O6	884.7833	$[M+NH_4]^+$	23.9

Lipidomics	TG	TG 54:3	TG 16:0_18:1_20:2	C57H104O6	884.7833	$[M+NH_4]^+$	23.9
Lipidomics	TG	TG 54:3	TG 18:0_18:1_18:2	C57H104O6	884.7833	$[M+NH_4]^+$	23.9
Lipidomics	TG	TG 54:3	TG 18:1_18:1_18:1	C57H104O6	884.7833	$[M+NH_4]^+$	23.9
Lipidomics	TG	TG 54:4	TG 16:0_18:1_20:3	C57H102O6	882.7676	$[M+NH_4]^+$	23.5
Lipidomics	TG	TG 54:4	TG 16:0_18:2_20:2	C57H102O6	882.7676	$[M+NH_4]^+$	23.5
Lipidomics	TG	TG 54:4	TG 16:1_18:1_20:2	C57H102O6	882.7676	$[M+NH_4]^+$	23.5
Lipidomics	TG	TG 54:4	TG 18:0_18:2_18:2	C57H102O6	882.7676	$[M+NH_4]^+$	23.5
Lipidomics	TG	TG 54:4	TG 18:1_18:1_18:2	C57H102O6	882.7676	$[M+NH_4]^+$	23.5
Lipidomics	TG	TG 54:5	TG 16:0_16:0_22:5	C57H100O6	880.7520	$[M+NH_4]^+$	23.2
Lipidomics	TG	TG 54:5	TG 16:0_18:2_20:3	C57H100O6	880.7520	$[M+NH_4]^+$	23.2
Lipidomics	TG	TG 54:5	TG 18:1_18:2_18:2	C57H100O6	880.7520	$[M+NH_4]^+$	23.2
Lipidomics	TG	TG 54:6	TG 16:0_16:1_22:5	C57H98O6	878.7363	$[M+NH_4]^+$	22.9
Lipidomics	TG	TG 54:6	TG 16:1_18:2_20:3	C57H98O6	878.7363	$[M+NH_4]^+$	22.9
Lipidomics	TG	TG 54:6	TG 18:1_18:2_18:3	C57H98O6	878.7363	$[M+NH_4]^+$	22.9
Lipidomics	TG	TG 54:6	TG 18:2_18:2_18:2	C57H98O6	878.7363	$[M+NH_4]^+$	22.9
Lipidomics	TG	TG 54:7	TG 16:1_16:1_22:5	C57H96O6	876.7207	$[M+NH_4]^+$	22.5
Lipidomics	TG	TG 54:7	TG 16:1_18:2_20:4	C57H96O6	876.7207	$[M+NH_4]^+$	22.5
Lipidomics	TG	TG 54:7	TG 18:2_18:2_18:3	C57H96O6	876.7207	$[M+NH_4]^+$	22.5
Lipidomics	TG	TG 56:5	TG 16:0_18:1_22:4	C59H104O6	908.7833	$[M+NH_4]^+$	23.8
Lipidomics	TG	TG 56:5	TG 18:0_18:1_20:4	C59H104O6	908.7833	$[M+NH_4]^+$	23.8
Lipidomics	TG	TG 56:5	TG 18:0_18:2_20:3	C59H104O6	908.7833	$[M+NH_4]^+$	23.8
Lipidomics	TG	TG 56:5	TG 18:1_18:1_20:3	C59H104O6	908.7833	$[M+NH_4]^+$	23.8
Lipidomics	TG	TG 56:5	TG 18:1_18:2_20:2	C59H104O6	908.7833	$[M+NH_4]^+$	23.8
Lipidomics	TG	TG 56:6	TG 16:0_18:1_22:5	C59H102O6	906.7676	$[M+NH_4]^+$	23.4
Lipidomics	TG	TG 56:6	TG 16:0_18:2_22:4	C59H102O6	906.7676	$[M+NH_4]^+$	23.4
Lipidomics	TG	TG 56:6	TG 18:0_18:2_20:4	C59H102O6	906.7676	$[M+NH_4]^+$	23.4
Lipidomics	TG	TG 56:6	TG 18:1_18:1_20:4	C59H102O6	906.7676	$[M+NH_4]^+$	23.4
Lipidomics	TG	TG 56:6	TG 18:1_18:2_20:3	C59H102O6	906.7676	$[M+NH_4]^+$	23.4
Lipidomics	TG	TG 56:7	TG 16:0_18:1_22:6	C59H100O6	904.7520	$[M+NH_4]^+$	23.1
Lipidomics	TG	TG 56:7	TG 16:0_18:2_22:5	C59H100O6	904.7520	$[M+NH_4]^+$	23.1

Lipidomics	TG	TG 56:7	TG 16:0_18:3_22:4	C59H100O6	904.7520	$[M+NH_4]^+$	23.1
Lipidomics	TG	TG 56:7	TG 16:1_18:2_22:4	C59H100O6	904.7520	$[M+NH_4]^+$	23.1
Lipidomics	TG	TG 56:7	TG 16:0_20:3_20:4	C59H100O6	904.7520	$[M+NH_4]^+$	23.1
Lipidomics	TG	TG 56:7	TG 18:1_18:1_20:5	C59H100O6	904.7520	$[M+NH_4]^+$	23.1
Lipidomics	TG	TG 56:7	TG 18:1_18:2_20:4	C59H100O6	904.7520	$[M+NH_4]^+$	23.1
Lipidomics	TG	TG 56:7	TG 18:1_18:3_20:3	C59H100O6	904.7520	$[M+NH_4]^+$	23.1
Lipidomics	TG	TG 56:7	TG 18:2_18:2_20:3	C59H100O6	904.7520	$[M+NH_4]^+$	23.1
Lipidomics	TG	TG 56:8	TG 16:0_18:2_22:6	C59H98O6	902.7363	$[M+NH_4]^+$	22.9
Lipidomics	TG	TG 56:8	TG 16:0_20:4_20:4	C59H98O6	902.7363	$[M+NH_4]^+$	22.9
Lipidomics	TG	TG 56:8	TG 18:1_18:2_20:5	C59H98O6	902.7363	$[M+NH_4]^+$	22.9
Lipidomics	TG	TG 56:8	TG 18:2_18:2_20:4	C59H98O6	902.7363	$[M+NH_4]^+$	22.9
Lipidomics	TG	TG 56:9	TG 16:0_18:3_22:6	C59H96O6	900.7207	$[M+NH_4]^+$	22.5
Lipidomics	TG	TG 56:9	TG 18:2_18:2_20:5	C59H96O6	900.7207	$[M+NH_4]^+$	22.5
Lipidomics	TG	TG 56:9	TG 18:2_18:3_20:4	C59H96O6	900.7207	$[M+NH_4]^+$	22.5
Lipidomics	TG	TG 58:10	TG 16:0_20:4_22:6	C61H98O6	926.7363	$[M+NH_4]^+$	22.6
Lipidomics	TG	TG 58:7	TG 18:0_18:2_22:5	C61H104O6	932.7833	$[M+NH_4]^+$	23.6
Lipidomics	TG	TG 58:7	TG 18:1_18:1_22:5	C61H104O6	932.7833	$[M+NH_4]^+$	23.6
Lipidomics	TG	TG 58:7	TG 18:1_18:2_22:4	C61H104O6	932.7833	$[M+NH_4]^+$	23.6
Lipidomics	TG	TG 58:8	TG 18:0_18:2_22:6	C61H102O6	930.7676	$[M+NH_4]^+$	23.3
Lipidomics	TG	TG 58:8	TG 18:1_18:1_22:6	C61H102O6	930.7676	$[M+NH_4]^+$	23.3
Lipidomics	TG	TG 58:8	TG 18:1_18:2_22:5	C61H102O6	930.7676	$[M+NH_4]^+$	23.3
Lipidomics	TG	TG 58:8	TG 18:2_18:2_22:4	C61H102O6	930.7676	$[M+NH_4]^+$	23.3
Lipidomics	TG	TG 58:8	TG 18:2_20:3_20:3	C61H102O6	930.7676	$[M+NH_4]^+$	23.3
Lipidomics	TG	TG 58:9	TG 18:1_18:2_22:6	C61H100O6	928.7520	$[M+NH_4]^+$	22.9
Lipidomics	TG	TG 58:9	TG 18:1_20:4_20:4	C61H100O6	928.7520	$[M+NH_4]^+$	22.9
Lipidomics	TG	TG 58:9	TG 18:2_18:2_22:5	C61H100O6	928.7520	$[M+NH_4]^+$	22.9
Lipidomics	TG	TG 58:9	TG 18:2_20:3_20:4	C61H100O6	928.7520	$[M+NH_4]^+$	22.9

Supplementary Table S2. Characteristics of the constructed unsupervised and supervised models. In models based on NAFLD groups pareto (PAR) scale was used.

**Model**	**Type**	**N**	**R^2X**	**R^2Y**	**Q^2**	**CV ANOVA**
**Plasma +ESI Disease groups**						
Control_NAFL_NASH_QC	PCA-X	46	0.827		0.632	
Control_NAFL_NASH	PCA-X	37	0.828		0.627	
Control_NAFL_NASH	PLS	37	0.509	0.452	0.207	
Control_NASH	OPLS-DA	27	0.41	0.613	0.428	1.22E-02
